# Identification of a Novel Human Papillomavirus, Type HPV199, Isolated from a Nasopharynx and Anal Canal, and Complete Genomic Characterization of Papillomavirus Species *Gamma*-12

**DOI:** 10.1371/journal.pone.0138628

**Published:** 2015-09-16

**Authors:** Anja Oštrbenk, Boštjan J. Kocjan, Lea Hošnjak, Jingjing Li, Qiuju Deng, Anja Šterbenc, Mario Poljak

**Affiliations:** 1 Institute of Microbiology and Immunology, Faculty of Medicine, University of Ljubljana, Ljubljana, Slovenia; 2 Key Laboratory of Carcinogenesis and Translational Research (Ministry of Education), Peking University Cancer Hospital & Institute, Beijing, China; University of Nebraska-Lincoln, UNITED STATES

## Abstract

The novel human papillomavirus type 199 (HPV199) was initially identified in a nasopharyngeal swab sample obtained from a 25 year-old immunocompetent male. The complete genome of HPV199 is 7,184 bp in length with a GC content of 36.5%. Comparative genomic characterization of HPV199 and its closest relatives showed the classical genomic organization of Gammapapillomaviruses (*Gamma*-PVs). HPV199 has seven major open reading frames (ORFs), encoding five early (E1, E2, E4, E6, and E7) and two late (L1 and L2) proteins, while lacking the E5 ORF. The long control region (LCR) of 513 bp is located between the L1 and E6 ORFs. Phylogenetic analysis additionally confirmed that HPV-199 clusters into the *Gamma*-PV genus, species *Gamma*-12, additionally containing HPV127, HV132, HPV148, HPV165, and three putative HPV types: KC5, CG2 and CG3. HPV199 is most closely related to HPV127 (nucleotide identity 77%). The complete viral genome sequence of additional HPV199 isolate was determined from anal canal swab sample. Two HPV199 complete viral sequences exhibit 99.4% nucleotide identity. To the best of our knowledge, this is the first member of *Gamma*-PV with complete nucleotide sequences determined from two independent clinical samples. To evaluate the tissue tropism of the novel HPV type, 916 clinical samples were tested using HPV199 type-specific real-time PCR: HPV199 was detected in 2/76 tissue samples of histologically confirmed common warts, 2/108 samples of eyebrow hair follicles, 2/137 anal canal swabs obtained from individuals with clinically evident anal pathology, 4/184 nasopharyngeal swabs and 3/411 cervical swabs obtained from women with normal cervical cytology. Although HPV199 was found in 1.4% of cutaneous and mucosal samples only, it exhibits dual tissue tropism. According to the results of our study and literature data, dual tropism of all *Gamma*-12 members is highly possible.

## Introduction

Human papillomaviruses (HPVs) are small, non-enveloped DNA viruses with a circular, double stranded genome of approximately 8,000 base pairs (bp). They are a group of highly divergent viruses that infect human epithelial cells, where they are implicated in the development of several benign and malignant neoplasms [1, 2]. HPVs are hierarchically classified into types, species and genera: HPV types that display less than 60% similarity in the L1 open reading frame (ORF) nucleotide sequence are classified to different genera, while viral species within the same genus share 60–70% similarity [1]. The taxonomy of papillomaviruses at the species level and above is verified by the International Committee for the Taxonomy of Viruses; however, HPV type designation and classification below the species level is maintained by the International HPV Reference Center at the Karolinska Institute, Stockholm, Sweden [3]. A consecutive number is assigned to a novel HPV type only after the following conditions are met: (i) the putative novel HPV type shows less than 90% nucleotide sequence identity with all known HPV types and (ii) a reference clone is deposited at the HPV reference center, where they re-clone and re-sequence the putative novel HPV type [1, 3]. As of May 30, 2015, two hundred and one different HPV types, ranging from HPV-1 to HPV-205, were officially recognized: 65 *Alphapapillomaviruses* (*Alpha*-PVs), 51 *Betapapillomaviruses* (*Beta*-PVs), 81 *Gammapapillomaviruses* (*Gamma*-PVs), 3 *Mupapillomaviruses* (*Mu*-PVs), and a single *Nupapillomavirus* (*Nu*-PV) (http://www.hpvcenter.se/html/refclones.html). Four previously recognized HPV types (HPV-46, HPV-55, HPV-64 and HPV-79) were recently re-classified as subtypes.

HPV types have traditionally been classified as mucosal or cutaneous on the assumption that HPV tissue tropism reflects the location from which the original isolate of the particular type was found [4, 5]. Accordingly, HPV types belonging to the *Alpha*-PVs are predominantly assigned as anogenital types and *Beta*-PVs, *Gamma*-PVs, *Mu*-PVs and *Nu*-PVs as cutaneous types [2, 4]. However, a growing body of evidence suggests that HPV types belonging to *Gamma*-PVs (the genus that has been growing most rapidly in recent years and is currently divided into 27 species) are ubiquitous and show much broader tissue tropism than previously thought [5], with reported detection sites ranging from healthy skin and various cutaneous lesions [6–9] to genital lesions [10] and oral [4] and nasal [11] mucosa. One recent report suggests that *Gamma*-PVs are also able to infect mucocutaneous sites such as the anal canal [12].

In the present study, we report a novel *Gamma*-PV type, HPV199, originally found in a nasopharyngeal swab sample obtained from a 25 year-old healthy individual. We fully characterized and phylogenetically evaluated the complete genome sequences of HPV199 and its closest relatives. The novel HPV type belongs to species *Gamma*-12, which so far contains only four officially recognized HPV types: HPV127, HPV132, HPV148 and HPV165 and 3 putative novel HPV types: CG2, CG3 and KC5 [3, 5]. The HPV199 genome organization was further investigated by comparison of the long control region, ORFs and protein functional domains with its closest relatives. The complete viral genome sequence of additional HPV199 isolate was determined from anal canal swab sample. Furthermore, an HPV199 type-specific RT-PCR was developed in order to test a representative collection of various HPV-associated clinical specimens to ascertain the tissue tropism of HPV199.

## Materials and Methods

### Identification of a partial HPV199 331 bp L1 gene sequence

In order to identify novel HPV types, stored DNA samples isolated from nasopharyngeal swabs were tested using different primer sets targeting a broad spectrum of HPV types belonging to the *Alpha*-PV, *Beta*-PV and *Gamma*-PV genera, as described previously [13–17]. Swabs were collected from 75 men and 100 women (age range 0–77 years, median age 28.0 years) with suspected whooping cough who had been referred for routine PCR testing for *Bordetella pertussis*. PCR products of the expected sizes were purified using a QIAquick PCR Purification Kit (Qiagen) and processed for sequence analysis and HPV type determination, as described previously [13]. In a nasopharyngeal swab obtained from a 25 year-old immunocompetent male, a partial HPV L1 gene sequence of 331 bp in length was obtained using the broad-range FAP6085F/FAP64 PCR protocol, as described previously [17], which did not correspond to any of the already established HPV types. This initial HPV199 sequence was deposited in the GenBank sequence database in November 2013, under accession number HG515499.

Total DNA from the original nasopharyngeal sample containing HPV199 was isolated using a QiAamp DNA Investigator Kit (Qiagen, Hilden, Germany), following a slightly modified protocol for isolation of DNA from surface and buccal swabs (Qiagen). Briefly, swabs were placed in a 1.5 ml microcentrifuge tube with 20 μl of proteinase K and 180 μl of buffer ATL and incubated for 15 min at 56°C. After incubation, 200 μl of buffer AL, 1 μl of carrier RNA (1 μg/μl) and 200 μl of ethanol (96–100%) were added to the samples, mixed thoroughly and incubated for 5 min at room temperature. Each sample was transferred to a QIAamp MinElute column and centrifuged for 1 min at 8,000 rpm. The columns were washed once with 500 μL of AW1-buffer, once with 700 μL of AW2-buffer and once with 700 μL of ethanol, and centrifuged each time for 1 min at 8,000 rpm. This was followed by centrifugation of a column for 3 min at 14,000 rpm. Bound DNA was eluted with 100 μL of ATE buffer and stored at—20°C until molecular analysis.

### PCR amplification of a full genome of HPV199

Based on the HPV199 L1 partial 331 bp sequence, a primer set KC82-LNG-F (5'- GGCAATAAGGGTGATTGTCCT-3', nt 5,694–5,714) and KC82-LNG-R (5'- TTTTTTACAAGGTTCAGCAACATC-3', nt 5,693–5,670), for the inverse long-range PCR was manually constructed using the BioEdit software package [18] and evaluated and corrected using the Netprimer program (http://www.premierbiosoft.com/netprimer/index.html). Prior to full-genome amplification of HPV199, sample DNA was subjected to rolling circle amplification (RCA) using an Ilustra TempliPhi^TM^ 100 Amplification Kit (GE Healthcare, Amersham, UK), as described previously [19]. The obtained RCA product was diluted in nuclease free water (ratio 1:100) and used in the subsequent PCR reaction.

A complete viral genome was amplified in several replicates using a Platinum Taq DNA Polymerase High Fidelity Kit (Invitrogen, Carlsbad, USA). The reaction mixture had a final volume of 25 μl and contained 5 μl of diluted RCA product, 2.5 μl of 10X High Fidelity PCR Buffer, 1 μl of 50 mM MgSO_4_, 200 μM of dNTPs, 0.5 U of Platinum Taq DNA Polymerase High Fidelity, 0.4 μM of each primer, and water. PCR-amplification was performed on a Veriti Thermal Cycler (Applied Biosystems, Foster City, USA) under the following conditions: 2 min of denaturation at 94°C, followed by 45 cycles of 30 s at 94°C, 30 s at 54°C and 8 min at 68°C, and a final elongation step of 7 min at 68°C. After amplification was completed, the reaction mixture was cooled to 8°C.

### Sequencing and cloning of the complete HPV199 genome

After amplification, the obtained PCR products were separated by gel electrophoresis and visible bands of the expected size were purified with a QIAquick PCR Purification Kit (Qiagen). One PCR product was selected to determine the viral full-genome sequence using a primer walking strategy at Peking University Cancer Hospital & Institute. Primers used for HPV199 whole genome sequencing are listed in S1 Table. This particular amplicon was additionally cloned into pCR-XL-TOPO plasmid vector using a TOPO XL PCR Cloning Kit (Invitrogen), according to the manufacturer’s instructions. After transformation of One Shot TOP10 chemically competent *E*. *coli* cells (Invitrogen) with HPV199 plasmids, the transformants were incubated overnight at 37°C on LB plates containing 50 μg/mL kanamycin. Bacterial colonies containing a fragment of the appropriate size were selected by colony PCR using FastStart PCR Master (Roche diagnostics) and M13 Forward (-20)/M13 reverse primers (Invitrogen), and grown overnight in LB kanamycin (50 μg/mL) medium. HPV199 plasmid DNA was extracted from 4 ml of bacterial culture using a QIAprep Spin Miniprep Kit (Qiagen), as instructed by the manufacturer. To avoid possible differences in nucleotide sequences of HPV199 PCR amplicons and plasmid clones, primer walking was repeated on one clone using identical primers (S1 Table). HPV199 plasmid DNA was sequenced in in-house sequence facilities using a Big Dye Terminator v3.1 Cycle Sequencing Kit (PE Applied Biosystems, Foster City, CA), as described previously [20], and analyzed on 3500 Series Genetic (PE Applied Biosystems, Foster City, CA).

### HPV199 genomic characterization

The complete nucleotide sequence of the HPV199 genome was assembled and edited with the Vector NTI Advance 11 program package (Invitrogen). ORFs of HPV199 were predicted by the ORF Finder Tool (http://www.ncbi.nlm.nih.gov/gorf/gorf.html) and verified with Vector NTI. The HPV199 genome organization was further investigated by comparing nucleotide alignments of individual genes of HPV199 and its closest relatives using the MEGA6 software package [21]. The detailed characterization of LCR, ORFs and protein functional domains of HPV199 and its closest relatives was done as described previously [22].

### Phylogenetic analysis

The complete genomes from all completely sequenced *Gamma*-PVs, including all putative novel types pending classification at the HPV reference center, were obtained from the Papillomavirus Episteme database (PaVE) [23] and aligned by the MUSCLE algorithm [24, 25] of the MEGA6 software package [21]. The first ATG of E6 was defined as the starting position for the sequence alignment, with the exception of HPV101 and HPV103, which lack E6 proteins [26]. The nucleotide sequences of two *Beta*-PVs, HPV5 and HPV8, were used to root the tree. All further phylogenetic analyses were performed with MEGA6. A General Time Reversible (GTR) model with five Gamma discrete categories (+G), with an allowance for the presence of invariant sites (+I), was determined by MEGA6 to be the best fitting nucleotide substitution model. In order to construct the phylogenetic tree, the maximum likelihood algorithm was used, with 1,000 bootstrap replicates. A graphic presentation of the phylogenetic tree was made with FigTree software v1.4.2 (http://tree.bio.ed.ac.uk/software/figtree/).

The phylogenetic relationship of HPV199 with other members of species *Gamma*-12 was analyzed using the EMBOSS Water Pairwise Sequence Alignment tool (http://www.ebi.ac.uk/Tools/psa/emboss_water/), which uses the Smith-Waterman algorithm to calculate the local alignment of individual sequences. With this method, we performed pairwise alignment of nucleotide and amino acid sequences for all seven viral proteins and the LCR region and calculated the percentage similarity. The phylogenetic relationship was further confirmed with the PASC tool at NCBI [27], which uses the Needleman-Wunsch algorithm to calculate the global alignment of sequences based on the complete genome. In order to determine whether two genomes exhibit similar degrees of homology across different regions of their genomes, the ratios of relatedness (L1:E1 ORF ratio) were calculated for all known and putative HPV types within *Gamma*-12 [28, 29].

### HPV199 type-specific real-time PCR

HPV199 type-specific primers and a probe for quantitative RT-PCR were designed within the HPV199 L2 gene, using on-line primer/probe design software (http://eu.idtdna.com/PrimerQuest/Home/Index). The designed primers KC82RTF (5'-CATTAGAGCGTGTAGCAACTCGT-3', nt 4,364–4,386) and KC82RTR (5'-TGCTCAAATTGTAATGTGACATCTC-3', nt 4,499–4,523) resulted in a PCR product of 160 bp in length. A 5’ FAM/ZEN-labeled Taqman probe KC82RTSR (5'-AAGGCGGGAAGGCTGAAG-3', nt 4,447–4,464) was additionally constructed to monitor the specific real-time amplification of the targeted genomic fragment. In order to avoid cross-reactivity with closely related HPV types, the selected primers and probes were analyzed using NCBI Blast and specificity was additionally confirmed by sequencing all HPV199 RT-PCR positive amplicons.

The reaction mixture of the optimized HPV199 RT-PCR assay had a total volume of 20 μl, containing 5 μl of DNA sample, 10 μl of 2X LightCycler 480 Probes Master (Roche Diagnostics), 0.5 μM of each primer and probe, and 4.4 μl of water. RT-PCR was carried out in a 96-well plate on a LightCycler 480 II RT-PCR Instrument (Roche) under the following cycling conditions: 10 min of denaturation at 94°C (ramp rate 4.4°C/s), followed by 45 amplification cycles at 95°C for 10 s (4.4°C/s), 60°C for 30 s (2.2°C/s), and 72°C for 1 s (4.4°C/s). The final step consisted of cooling the reaction mixture to 40°C (2.2°C/s) with a 30 s hold. The fluorescent signal was measured on the FAM (465–510 nm) channel at the end of each step, at 72°C.

The analytical sensitivity of the assay was determined as described previously [30] by testing triplicates of a 10-fold dilution series of HPV199 reference plasmid, ranging from 1x10^9^ to 1x10^0^ DNA copies/reaction, and the detection limit was established to be at least ten viral copies. The standard curve of the assay was characterized by a high correlation coefficient (R^2^ = 0.999), high amplification efficiency (102.3%) and a dynamic range of eight orders of magnitude, enabling reliable discrimination of 10 to 10^9^ DNA copies/reaction. For each sample panel tested, several negative RT-PCR controls consisting of water were included to check for possible amplicon carry-over contamination.

### Determination of the tissue tropism of HPV199

To determine the tissue tropism of HPV199, a representative collection of various HPV-associated clinical specimens was tested with a HPV199 type-specific RT-PCR assay. In total, 916 samples obtained from the same number of immunocompetent individuals were tested: 76 tissue samples of histologically confirmed common warts, 108 samples of eyebrow hair follicles, 137 anal canal swabs obtained from individuals with clinically evident anal pathology, including anal warts, hemorrhoids and anal fissure, 184 nasopharyngeal swab samples and 411 cervical swabs obtained from women with normal cervical cytology. Total DNA was isolated from all samples using various, specimen-adjusted protocols, as described in detail previously [13, 31–33]. All clinical specimens were tested for human beta-globin to assess cell adequacy and the quality of the isolated DNA, and had to be positive to be included in the analysis. The presence of human beta-globin in cervical and anal swabs was determined by the internal control of the Linear Array HPV Genotyping Test (Linear Array; Roche Molecular Diagnostics, Pleasanton, CA), whereby an additional primer pair targeted 268 bp of the human beta-globin gene. The presence of the human beta-globin in all other clinical specimens was determined using a slightly modified RT-PCR, targeting a 150 bp long fragment of the human beta-globin gene [34].

### Ethics Statement

The present study was conducted in accordance with the Helsinki Declaration. All DNA samples tested for the presence of HPV199 DNA were obtained from our past or ongoing studies [13, 31, 33, 35] and none of the individuals were sampled solely for the objective of the present study. All studies from which samples for HPV199 testing were used have been approved by the National Medical Ethics Committee at the Slovenian Ministry of Health of the Republic of Slovenia (consent numbers as follows: 131/06/07, 45/04/07, 83/11/09, 109/08/12 and 63/10/13). All involved patients and individuals provided standardized written informed consents allowing testing for a broad range of HPVs for research purposes. In order to keep patient identities confidential, all samples were coded and only clinicians were able to link an individual’s identity with the assigned study number and HPV199 results. The Institutional Review Board of the Institute of Microbiology and Immunology, Faculty of Medicine, University of Ljubljana, specifically approved the protocol of the present study and the use of stored DNA samples for the HPV199 testing.

## Results and Discussion

HPV199 was originally found in a nasopharyngeal swab sample obtained from a 25 year-old immunocompetent male with a clinical suspicion of *Bordetella pertussis* infection. A partial 331 bp L1 sequence (Acc. no HG515499), suggesting the presence of a putative novel *Gamma*-PV type, was initially obtained with FAP6085F/FAP64 primers [15, 16], using a previously published PCR protocol [17]. Nasopharyngeal swab sample additionally tested positive for HPV5, HPV8 and HPV12 with primer sets targeting a broad spectrum of HPV types belonging to the *Alpha*-PV, *Beta*-PV and *Gamma*-PV genera [15–17]. The complete viral genome was amplified using HPV199 type-specific inverse long range PCR and cloned into a plasmid vector, and sequenced using a primer walking strategy. In order to determine possible artifacts in the cloned viral genome, the corresponding amplicon used for HPV199 cloning was additionally completely sequenced. Namely, it has been shown that alterations during the cloning process can occur, indicating the possibility that the cloned sequence is not an authentic copy of the genomic sequence [36, 37]. Since 100% identity in the cloned HPV199 DNA sequence and viral genomic HPV199 DNA was determined by both methods, the generation of a cloning artifact due to the cloning process is highly unlikely [36–38]. In May 2014, the reference clone and corresponding nucleotide sequence were submitted to the Human Papillomavirus Reference Center at the Karolinska Institute in Sweden, where its nucleotide sequence was reconfirmed and assigned the official number HPV199 in July 2014 (http://www.hpvcenter.se/html/refclones.html). The complete genome sequence was deposited in GenBank under accession number KJ913662.

In order to confirm uniqueness of the HPV199 we determined a complete nucleotide sequence of one additional HPV199 DNA isolate. The additional HPV199 complete viral genome was amplified using HPV199 type-specific inverse long range PCR and sequenced using primer walking strategy from anal canal swab sample obtained from 39-year old immunocompetent male. The complete viral genome of the second HPV199 DNA isolate exhibits 99.4% nucleotide identity with the original HPV199 isolate, suggesting new variant of the novel HPV199. The complete genome sequence of this isolate was deposited in GenBank under accession number KT372348.

**Fig 1:**
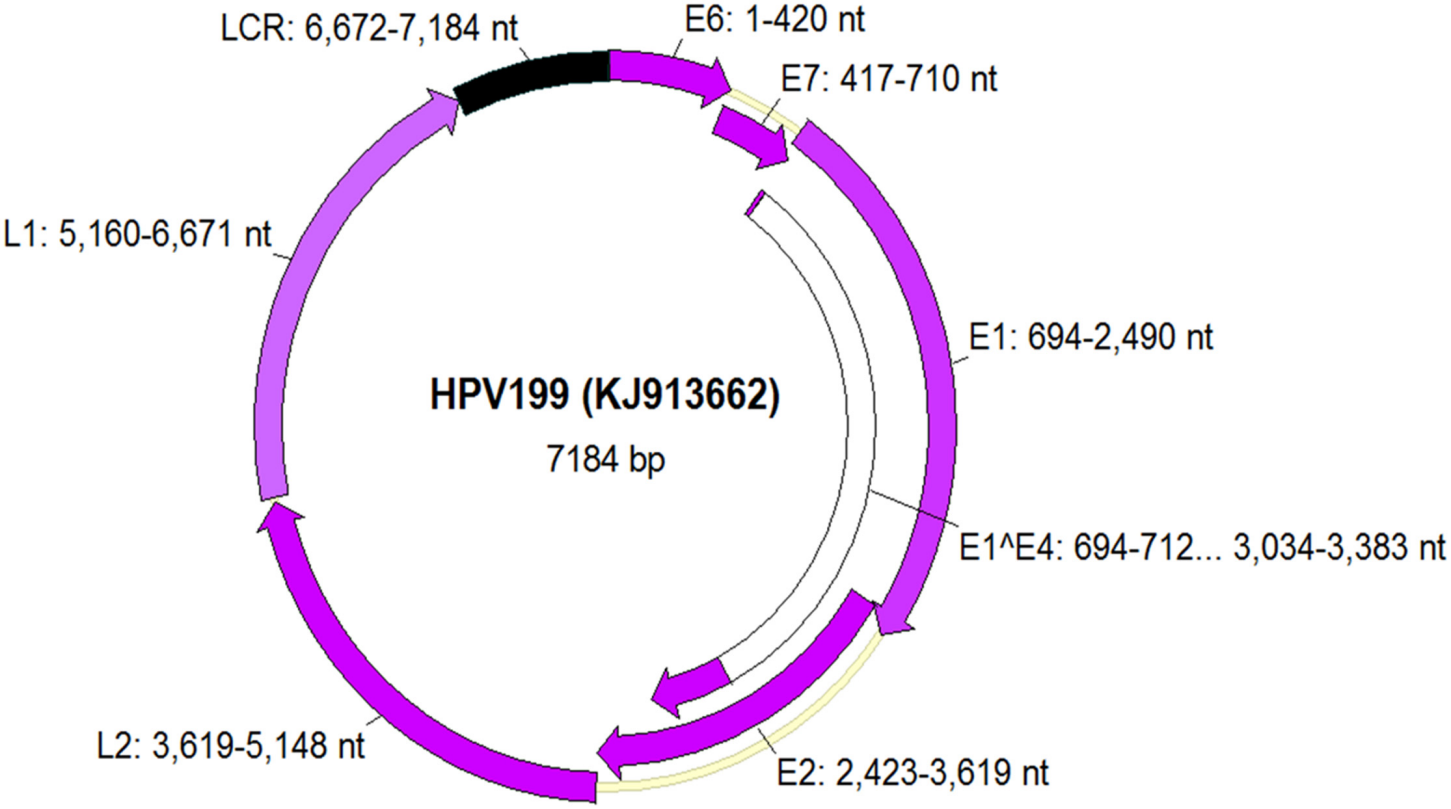
Genomic organization of HPV199 showing genomic positions of viral genes E6, E7, E1, E2, E4, L1, L2 and the non-coding long control region (LCR) located between L1 and E6.

As shown in Fig 1, the complete genome of HPV199 is 7,184 bp in length with a GC content of 36.5%. It demonstrates the typical genomic organization of *Gamma*-PVs, with the seven classical major open reading frames (ORFs), encoding five early (E1, E2, E4, E6 and E7) and two late proteins (L1 and L2). As expected for *Gamma*-PVs, none of the other small ORFs identified in the genome of HPV199 showed significant similarity to known E5 proteins [39].

**Fig 2:**
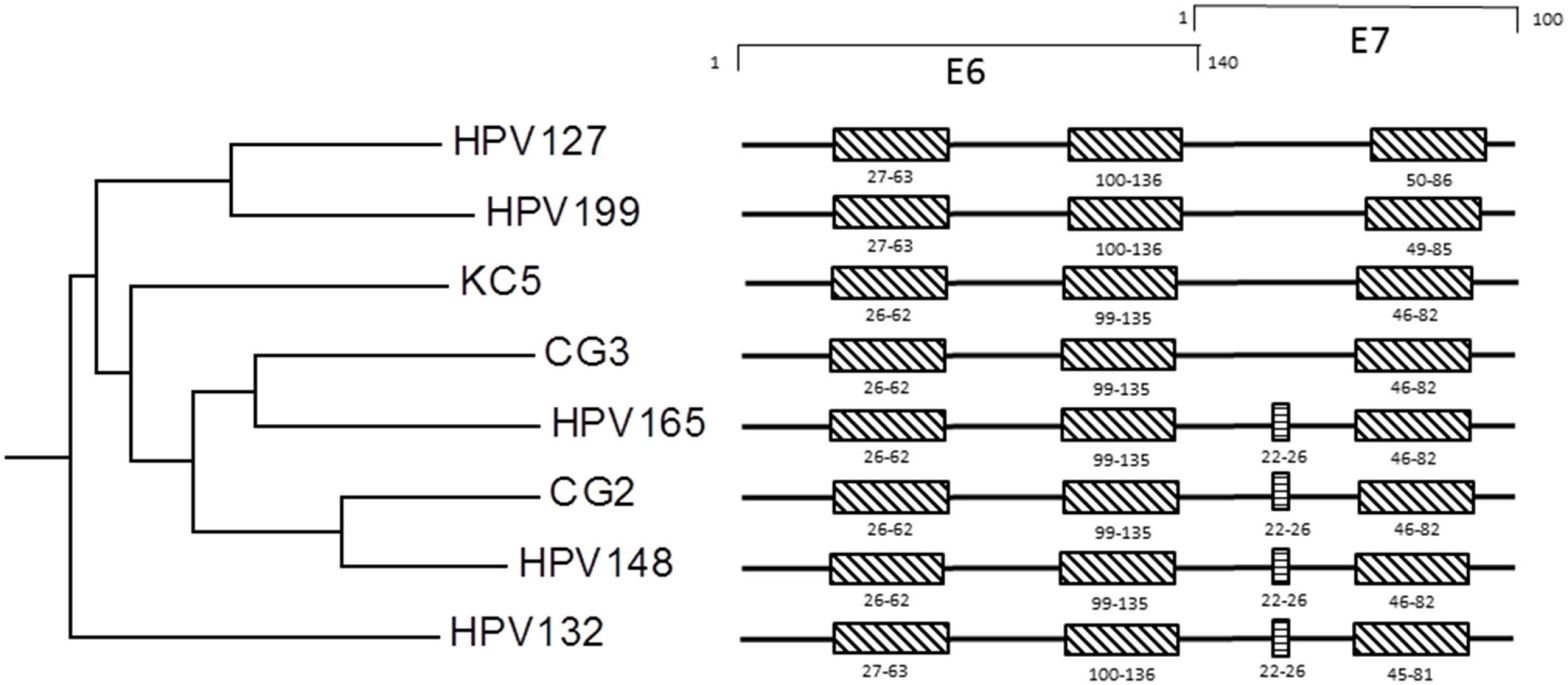
Localization of zinc-finger domains and binding domain for conserved retinoblastoma tumor suppressor protein (pRb) in E6 and E7 ORF of HPV199 and phylogenetically related HPV types. The E6 and E7 ORFs of all HPV types belonging to *Gamma*-PV species 12 contain two and one zinc-finger domains (CxxC(x)29CxxC), respectively. The pRB binding motif (LxCxE) was identified only in the E7 ORF of HPV165, CG2, HPV148 and HPV132. Locations of zinc-finger domains are marked as boxes with oblique lines and locations of pRb binding domains as boxes with horizontal lines.

The first position of the nucleotide sequence of the whole genome of HPV199 corresponds to the first ATG of the putative E6 protein, which contains two conserved zinc-finger domains (CxxC(x)_29_CxxC) that are separated by 36 amino acids. The putative E7 protein that is located downstream of E6 ORF contains one zinc-finger domain (Fig 2). As additionally shown in Fig 2, these particular domains were also identified in E6 and E7 proteins of all other members of the species *Gamma*-12. Zinc-finger domains seem to be essential structures in the formation of multimerized protein complexes [40–42]. As with several other HPV types, HPV199 E7 protein contains no binding domain (LxCxE) for the conserved retinoblastoma tumor suppressor protein (pRB). This conserved motif is necessary and sufficient for the association between E7 and pRB [43], although it has been shown that some types lacking the LxCxE motif cluster together in a phylogenetic tree, suggesting the motif was lost in the ancestral HPV of the cluster [44]. On the other hand, our analysis showed that four members of the same species (HPV132, HPV148, HPV165 and CG2) contain a pRB binding motif in the N-terminal part of the E7 protein (Fig 2). These results are consistent with results published earlier for HPV132 and HPV148 [45].

One conserved ATP-binding site of the ATP-dependent DNA helicase with a consensus sequence (GXXXXGK(T/S) was identified at aa 426–433 in the largest protein of HPV199 E1, with 598 amino acids [46]. This particular domain was additionally identified in E1 proteins of all other members of the species *Gamma*-12 (S1 Fig). When expressed, E1 accumulates primarily in the nucleus of transfected cells. Nevertheless, studies on HPV11, HPV31 and BPV1 E1 have demonstrated that, although this protein is predominantly found in the nucleus at the steady-state, it can shuttle between the nucleus and cytoplasm via distinct nuclear localization and export signals [47].

Consequently, a typical bipartite nuclear localization signal (NLS), composed of two clusters of aa (KRK and KRRL) separated by 27 aa, was identified at the HPV199 E1 n-terminal part at codons 80–113 (nt, 928–1029) (S2 Fig). Similarly, a leucine-rich nuclear export signal (NES) (L-X_(2–3)_-L-X_2_-(L/I/V)-X-(L/I)), typically recognized by the cell Crm1 exportin [47], was identified within the NLS region of the HPV199 E1 protein at codons 94–103 (nt, 970–999) (S2 Fig). As shown in S2 Fig, both NLSs and NESs were also successfully identified in all relatives of HPV199, with some minor differences in the aa composition.

No conserved leucine zipper domain (L-X_6_-L-X_6_-L-X_6_-L) is present in the carboxyterminal part of the putative E2 protein of HPV199. A highly conserved NLS motif (RKRXR), which is present in many of the *Alpha*-HPVs and which in HPV6 and HPV11 promotes nuclear localization and association of the E2 proteins with the nuclear matrix [48], was identified at codons 231–235 (S3 Fig). As additionally shown in S3 Fig, the same conserved or slightly modified NLS motif was identified in E2 proteins of all other members of the species *Gamma*-12.

The HPV199 E4 ORF is typically positioned within the E2 ORF (nt, 2,997–3,383) and has its own start codon. However, due to the presence of the characteristic donor (AAG/GUASNR) and acceptor (GUYACYAG/YU) RNA splicing sites in E1 and E2 ORFs, respectively, [5, 49] it is most likely that the E4 protein is translated from a spliced mRNA, containing the first few codons of the E1 ORF joined to the E4 sequence (Fig 1, E1^E4, coding sequence consisting of genomic positions nt 694…712 and 3,034…3,383), as described previously for other HPV types [22, 50]. We observed a typically high proline content in the E1^E4 protein, with 20 proline residues out of 128 amino acids (15.6%).

The HPV199 late genomic region encodes two structural proteins: the major (L1) and minor (L2) capsid proteins. Assembly of PV virions occurs in the cell nucleus and a highly conserved polybasic patch at (or very near) the C-terminus of L1 and L2 has been identified as directing their import into the nucleus [51–54]. Consequently, NLS-like signals were identified at the C-terminal part of L1 and L2 proteins of HPV199 and all other members of the species *Gamma*-12 (S4 and S5 Figs) [51, 52]. In addition, the N-terminal part of the L2 protein of all *Gamma*-12 HPVs (except HPV165) contains a highly conserved furin cleavage motif (R-X-K/R-R) (S6 Fig). The cleavage of the HPV L2 protein by cellular furin protease results in viral capsid conformational changes, leading to internalization of the virions into the basal cells to deliver the viral genome to their nucleus [53]. HPV165 probably has a differently shaped capsid, which does not require a second conformational change and therefore lacks the furin cleavage motif. Following viral dissociation, the viral DNA must escape the late endosome to be able to travel to the nucleus [53]. It has recently been suggested that a transmembrane domain located near the N-terminus of the L2 protein probably aids vesicular compartment escape of the L2/HPV-DNA complex [53, 55]. In line with this observation, the L2 transmembrane domain-like aa sequence (IVYFGGLGIGSGKGSG) was identified in the HPV199 L2 protein at codons 49–67 (nt, 3,763–3,819) (S7 Fig). As shown in S7 Fig, this domain was further identified in L2 of all relatives of HPV199, with some minor differences in the aa composition. Lastly, at its 5’ end, the HPV199 L2 ORF additionally contains a putative polyadenilation site (AATAAA; nt, 3,709–3,714) that is necessary for processing viral early gene transcripts. This particular genetic signature was further identified at the same 5’ location within the L2 gene of all other *Gamma*-12 species members.

The HPV199 long control region (LCR) is positioned between L1 and E6 ORFs at nucleotide positions 6,672 to 7,184 (513 bp) and contains four consensus palindromic E2-binding sites (ACC-N_6-7_-GGT; nt 6,916–6,927, nt 6,957–6,969, nt 7,064–7,075, nt 7,133–7,144), a putative polyadenilation site (AATAAA, nt 6,767–6,772) for L1/L2 gene transcripts, a putative E1 binding site (consensus sequence: CTCATAGTTGCCAACTATTAT, nt 7,093–7,113), probably representing the origin of replication (S8 Fig) [56], and binding sites for transcriptional regulatory factors such as AP-1, NF-1, Sp1, TFIID and C/EBP [56, 57]. A putative TATA box (TATAAA) of the E6 promoter was identified 31 nt upstream of the first start codon of the E6 ORF [56, 57]. The same characteristics were examined in all other *Gamma*-12 species members and they exhibited similar genetic features of LCR as HPV199. The genetic characteristics of the LCR genomic region of all current and putative members of the species *Gamma*-12 are summarized in S2 Table.

**Fig 3:**
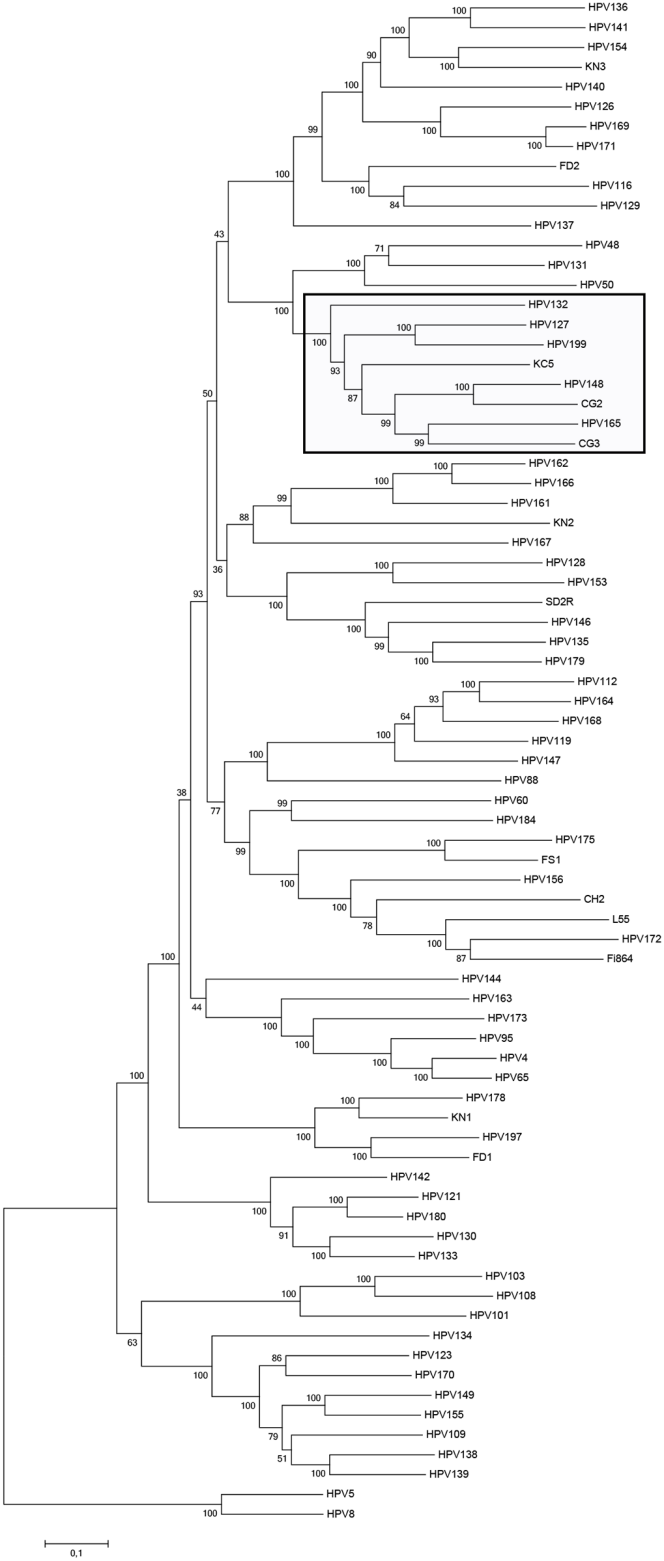
Phylogenetic analysis of HPV199. The complete HPV genome sequences of 75 officially recognized HPV types belonging to the genus *Gamma*-PV and all putative *Gamma*-PV types were obtained and aligned and a maximum likelihood phylogenetic tree was constructed. The nucleotide sequences of two *Beta*-PVs, HPV5 and HPV8, were used to root the tree. The numbers in each branch are bootstrap support values and are given as percentages. Species *Gamma*-12 is indicated by a black box.

Phylogenetic analysis revealed that HPV199 clusters into the *Gamma*-PV genus, species *Gamma*-12, which additionally contains HPV127, HV132, HPV148, HPV165, and three putative HPV types: KC5, CG2 and CG3 (Fig 3). An identical evolutionary relationship was observed in the phylogenetic tree based solely on the L1 nucleotide sequences. According to the topology of the constructed phylogenetic tree and PACS full-genome global alignment, HPV199 is most closely related to HPV127, with 77% nucleotide identity in the entire L1 region and 73.21% in the entire genome. Further analysis additionally confirmed HPV127 as the closest relative of HPV199, with the highest similarities in all compared viral ORFs and proteins (Table 1). As shown in Table 1, pairwise comparison of the novel HPV199 with the remaining members of *Gamma*-12 identified nucleotide similarities of the L1 ORF, with values ranging from 67.8–69.5%. The ratios of relatedness (L1:E1 ratio) were calculated within *Gamma*-12 members and are shown in Table 1. Values near 1.0 indicate that genomes within this species exhibit similar degrees of homology across different regions of their genomes [28, 29], thereby confirming their close phylogenetic relationship.

**Table 1:** Nucleotide and amino acid sequence percentage similarities between individual genes (E6, E7, E1, E2, E4, L2 and L1) and the long control region (LCR) of HPV199 and closest HPV types.

HPV types (*Gamma*-12)		Pairwise similarity with HPV199 (%)								L1:E1 ratio
		E6	E7	E1	E2	E4	L2	L1	LCR	
HPV127	nt	83.2	74.6	78.6	76.5	76.7	64.6	77.0	50.0	0.980
	aa	92.0	83.5	85.9	82.6	71.7	74.0	90.4	-	1.052
HPV132	nt	60.2	62.0	72.8	69.0	65.9	60.4	68.6	56.2	0.942
	aa	72.1	64.6	82.1	75.0	66.7	69.6	79.4	-	0.967
HPV148	nt	65.0	61.7	71.9	66.7	64.6	61.8	68.1	53.2	0.947
	aa	70.5	76.0	79.4	72.1	58.3	70.4	81.6	-	1.028
HPV165	nt	72.0	65.6	72.0	65.9	67.5	59.2	68.2	49.8	0.947
	aa	83.0	76.8	79.0	70.7	60.8	68.0	78.3	-	0.991
CG2	nt	66.8	58.5	70.8	66.0	66.7	60.2	67.9	53.5	0.959
	aa	70.9	75.0	79.8	70.4	58.3	72.1	81.1	-	1.016
CG3	nt	69.0	63.2	73.2	64.6	64.4	60.0	67.8	52.0	0.926
	aa	79.3	78.1	82.1	72.3	59.8	68.9	79.9	-	0.973
KC5	nt	68.5	68.2	74.6	68.0	67.4	61.2	69.5	53.9	0.932
	aa	78.5	74.5	84.1	72.6	63.4	71.7	82.8	-	0.985

Recent studies have detected *Gamma*-PVs at various sites, including healthy skin and various cutaneous lesions [6–9] and genital [10], anal [12], oral [4] and nasal [11] mucosa, suggesting that the original perception of *Gamma*-PVs as cutaneous HPV types was too narrow [5]. In contrast, members of *Gamma*-12 have so far been found predominantly in healthy skin samples and skin warts [5]. Specifically, HPV127, HPV132 and HPV165 were originally detected in skin swab samples obtained from healthy individuals, indicating that these viruses are probably skin commensals [9, 58, 59]. Additionally, recent metagenomic analysis using shotgun sequencing approach identified three *Gamma*-12 members HPV127, HPV132 and HPV148 in three, four and seven skin samples, obtained from healthy individuals, respectively [8]. In the same study HPV148 was also found in a single oral mucosa sample obtained from a healthy individual [8]. In recently published study, HPV132 and HPV148 were occasionally detected in anal samples obtained from HIV-positive and HIV-negative men who have sex with men [60]. On the other hand, the persistence of HPV132 and HPV148 in common skin warts and histologically confirmed actinic keratotic lesions, respectively, suggests that some members of *Gamma*-12 may be implicated in the development of benign and precancerous lesions in immunosuppressed individuals [45]. Three putative HPV types (CG2, CG3, KC5) that were preliminary classified into *Gamma*-12 [5], have been found in normal skin, while CG2 was additionally found in a skin sample of a female renal transplant recipient [7, 59, 61].

In order to assess HPV199 tissue tropism, various clinical specimens representing known sites of HPV infection were tested using an HPV199 type-specific RT-PCR assay (Table 2). The sample collection included cutaneous samples, particularly eyebrow hair follicles, which are a known reservoir of *Gamma*-PVs [9, 62] and common warts, which are cutaneous lesions with a productive HPV infection and which might be occasionally induced by *Gamma*-PVs [22]. Additionally, anal canal swabs, nasopharyngeal swabs and cervical swabs were tested to determine the prevalence of HPV199 in mucocutaneous and mucosal epithelia. As shown in Table 2, of 916 clinical samples tested, HPV199 DNA was detected in a total of 13 samples (1.4%), indicating a low overall prevalence of HPV199 across different human epithelia, similar to other *Gamma*-PVs [5, 22, 63]. The presence of other HPV types which were previously determined with several in-house and commercial HPV broad-range PCR protocols in our past or ongoing studies [13, 31, 33, 35] in all 916 clinical specimens used to determine HPV199 tissue tropism is summarized in S3 Table.

**Table 2:** Detection of HPV199 in 916 clinical samples using an HPV199 type-specific RT-PCR assay.

Tissue type	Anatomical location (sample type)	Total number of samples tested	Number of HPV199 positive samples	Prevalence of HPV199
Cutaneous	Common warts (tissue)	76	2	2.6%
	Eyebrows (hair follicles)	108	2	1.9%
Mucocutaneous	Anal canal (swabs)	137	2	1.5%
Mucosal	Nasopharynx (swabs)	184	4	2.2%
	Cervix (swabs)	411	3	0.7%
Total		916	13	1.4%

Although we detected HPV199 in two out of 76 (2.6%) common warts tested, we were not able to establish an etiological link between the presence of HPV199 and the development of these two common warts, due to the detection of other established common wart-associated HPV types, particularly HPV2, in both warts. However, data from some studies have shown that *Gamma*-PVs can be etiologically linked with sporadic cases of common warts and actinic keratosis, especially in immunosuppressed patients [22, 45, 64–67], so further studies are needed to determine the potential clinical significance of HPV199. HPV199 was further detected in two out of 108 (1.9%) samples of eyebrow hair follicles, confirming accumulating data about hair follicles as an important reservoir of various HPV types from different HPV genera [13, 22, 35, 68–70]. Additionally, JL and QD recently identified in a skin sample a partial L1 sequence 252 bp in length, with a 99% nucleotide identity to HPV199 (Acc. No KC752084) [17] but all attempts to clone HPV199 from this sample failed.

Since HPV199 was originally detected in the nasopharynx, a total of 184 nasopharyngeal swabs were additionally tested and four (2.2%) tested HPV199 positive. Our data are consistent with a recently published report indicating that *Gamma*-PVs exhibit tropism for nasal mucosa [11]. Although a relatively high prevalence of *Beta*-PV and *Gamma*-PV types was observed in this particular study, the potential clinical significance of *Beta*-PV and *Gamma*-PV types in the nasal region remains unknown [11].

HPV199 was also detected in two (1.5%) out of 137 anal canal swabs tested. Similar results were reported in a recently published study in which the diversity of HPV in the male anal canal was investigated and a broad spectrum of HPV types belonging to the *Alpha*-PV, *Beta*-PV and *Gamma*-PV genera was identified [12]. Although the prevalence and type-specific distributions of *Gamma*-PV (5.3%) were significantly lower than in other HPV genera, this area requires further research.

To the best of our knowledge, the prevalence of *Gamma*-PVs in cervical samples obtained from women with normal cervical cytology had not been evaluated prior to this study. In the initial testing of 96 cervical swab samples obtained from women with normal cervical cytology, one sample tested positive for the presence of HPV199. Since none of the published reports have previously detected any member of *Gamma*-12 in the cervix, a larger number (total of 411 samples) of cervical samples were additionally tested, and two more samples tested HPV199 positive, producing a total HPV199 prevalence of 0.7%. So far, only a few HPV types belonging to *Gamma*-PV have been found in the cervix [5, 8, 29, 71]. Three related HPV types: HPV101, HPV103 and HPV108, all belonging to *Gamma*-6, were previously found in samples of high-grade cervical intraepithelial neoplasia, normal genital mucosa and low-grade cervical intraepithelial neoplasia, respectively [29, 71] but a possible etiological link between HPV infection and the development of a cervical intraepithelial lesion has been established only for HPV108 [71]. A recent metagenomics analysis also revealed the presence of *Gamma*-PV in the anogenital region, more specifically vagina [8]. Thus, according to the results of our study and literature data, dual tropism of all *Gamma*-12 members is highly possible.

It has been shown previously that HPV types belonging to *Gamma*-12 can establish a persistent infection in immunosuppressed individuals [22, 45]. In our study, additional samples were available only for three HPV199 positive individuals but all three follow-up samples, obtained 35, 38 and 38 months after initial testing, respectively, tested HPV199 negative. Due to the low HPV199 initial prevalence and few available follow-up samples, we cannot draw a final conclusion about HPV199 persistence ability, so further studies on a larger number of samples are needed.

## Conclusions

A novel HPV type, HPV199, was initially identified in a nasopharyngeal swab sample and successfully characterized. The complete genome of HPV199 has a length of 7,184 bp and contains five early (E1, E2, E4, E6 and E7) and two late (L1 and L2) ORFs, but no E5 ORF, a genomic organization typical of other *Gamma*-PVs. Comparative characterization of HPV199 and its closest relatives and phylogenetic analysis confirmed that HPV-199 clusters into the *Gamma*-PV genus, species *Gamma*-12, additionally containing HPV127, HV132, HPV148, HPV165, and three putative HPV types: KC5, CG2 and CG3. The complete viral genome of additional HPV199 isolate was sequenced from anal canal swab sample. Two independent HPV199 complete viral sequences exhibit 99.4% nucleotide identity. To the best of our knowledge, this is the first member of *Gamma*-PV with complete nucleotide sequences determined from two independent clinical samples. Overall, HPV199 was found in 1.4% of cutaneous and mucosal samples tested only, but it exhibits dual tissue tropism. According to the results of our study and literature data, dual tropism of all *Gamma*-12 members is highly possible.

## Supporting Information

S1 Fig Conserved ATP-binding site of the ATP-dependent DNA helicase with consensus sequence (GXXXXGK(T/S) identified in E1 proteins of all *Gamma*-12 members. Consensus sequences are indicated by a red box.
(TIF)

S2 Fig Typical bipartite nuclear localization signal (NLS) and nuclear export signal (NES) identified in E1 proteins of all *Gamma*-12 members. NLS, composed of two clusters of aa (KRK and KRRL), is indicated with black boxes and NES with a red box.
(TIF)

S3 Fig Highly conserved nuclear localization signal (NLS) motif (RKRXR) in E2 proteins of all *Gamma*-12 members. A conserved or slightly modified NLS motif is indicated with red box.
(TIF)

S4 Fig Nuclear localization-like signals (NLS-like) at C-terminal part of L1 proteins of all *Gamma*-12 members. NLS-like signals are indicated with a red box.
(TIF)

S5 Fig Nuclear localization-like signals (NLS-like) at the C-terminal part of L2 proteins of all *Gamma*-12 members. NLS-like signals are indicated with a red box.
(TIF)

S6 Fig Highly conserved furin cleavage motif (R-X-K/R-R) at the N-terminal part of L2 proteins of all *Gamma*-12 members. Furin cleavage motifs are indicated with a red box.
(TIF)

S7 Fig L2 transmembrane domain-like aa sequence in L2 proteins of all *Gamma*-12 members. L2 transmembrane domain-like sequences with some minor differences in aa composition are indicated with a red box.
(TIF)

S8 Fig Putative E1-binding sites identified in the long control region (LCR) of all Gamma-12 members. A total of 144 HPV reference sequences was obtained from the Papillomavirus Episteme database (PaVE) and aligned with the Pro-Coffee algorithm of the T-coffee multiple sequence alignment package (http://tcoffee.crg.cat/apps/tcoffee/do:procoffee). The obtained alignment was visualized with the JalView 2.8.1 software package and colored based on the percentage identify option. The E1-binding site sequence logo was derived from the alignment and generated with WebLogo 2.8.2 using the web server at http://weblogo.berkeley.edu/logo.cgi.
(TIF)

S1 Table Primers used for HPV-199 whole genome sequencing using a primer walking strategy. (DOCX)

S2 Table Summary of genetic features of the LCR genomic region of current and putative members of species *Gamma*-12. (DOCX)

S3 Table HPV types determined in 916 various clinical specimens using an HPV199 type-specific real-time PCR assay and different primer sets targeting a broad spectrum of HPV types belonging to *Alpha*-PV, *Beta*-PV and *Gamma*-PV. (DOCX)
