# Supplementary figures and images for: Identification of a Novel Human Papillomavirus, Type HPV199, Isolated from a Nasopharynx and Anal Canal, and Complete Genomic Characterization of Papillomavirus Species Gamma-12
Source: PLoS One. 2015 Sep 16;10(9):e0138628. doi: 10.1371/journal.pone.0138628 (PMC4574437; doi:10.1371/journal.pone.0138628)

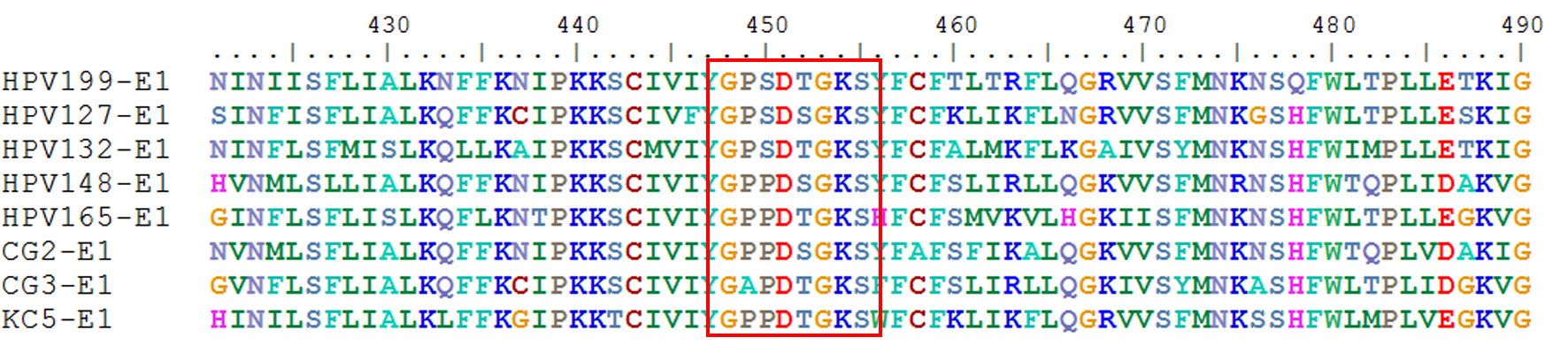

Supplement: S1 Fig — Consensus sequences are indicated by a red box. (TIF) [file pone.0138628.s001.tif]

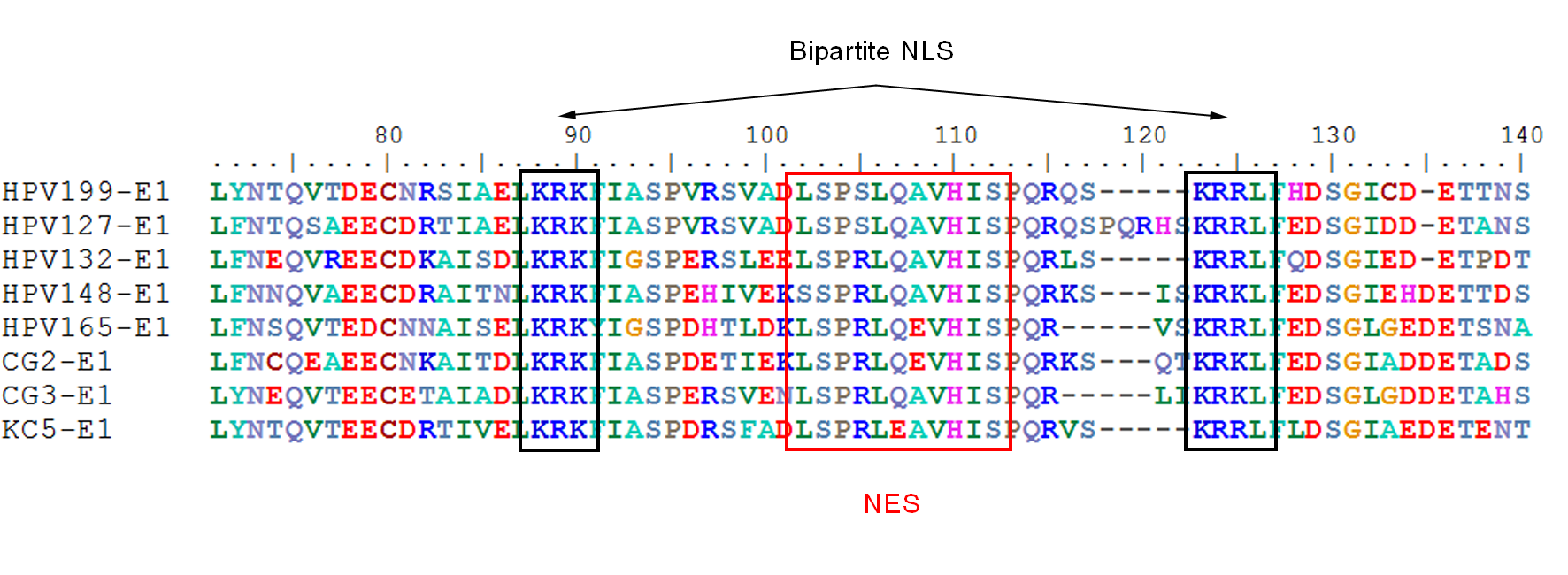

Supplement: S2 Fig — NLS, composed of two clusters of aa (KRK and KRRL), is indicated with black boxes and NES with a red box. (TIF) [file pone.0138628.s002.tif]

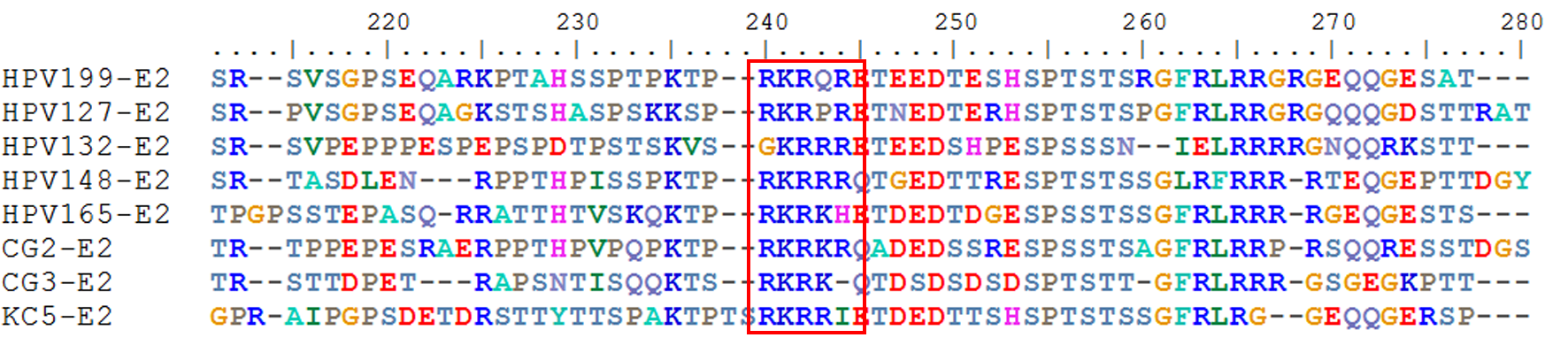

Supplement: S3 Fig — A conserved or slightly modified NLS motif is indicated with red box. (TIF) [file pone.0138628.s003.tif]

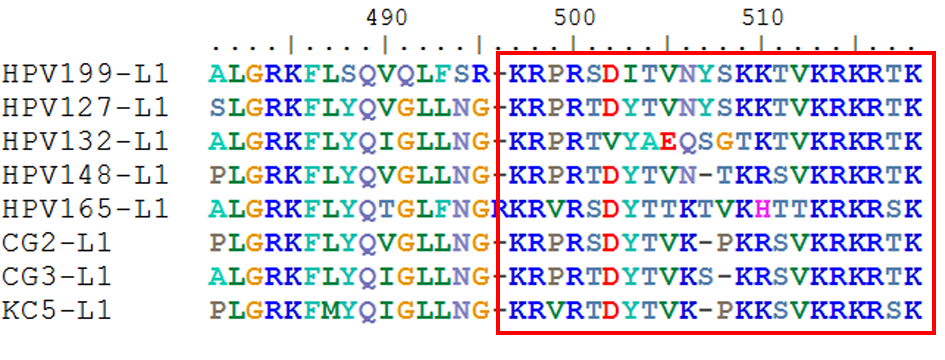

Supplement: S4 Fig — NLS-like signals are indicated with a red box. (TIF) [file pone.0138628.s004.tif]

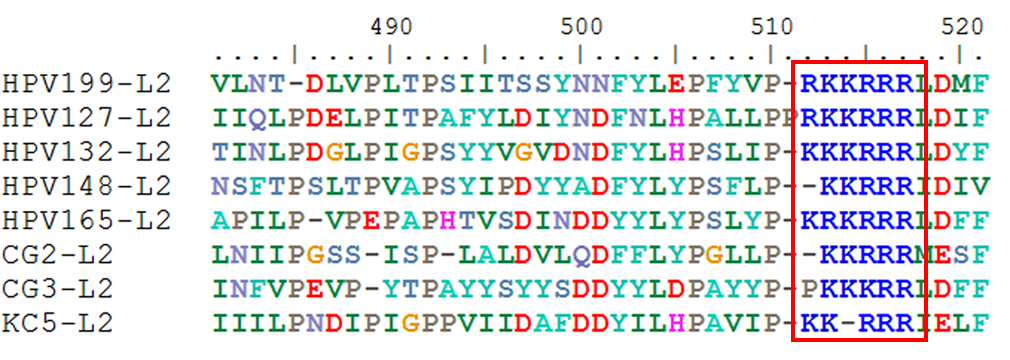

Supplement: S5 Fig — NLS-like signals are indicated with a red box. (TIF) [file pone.0138628.s005.tif]

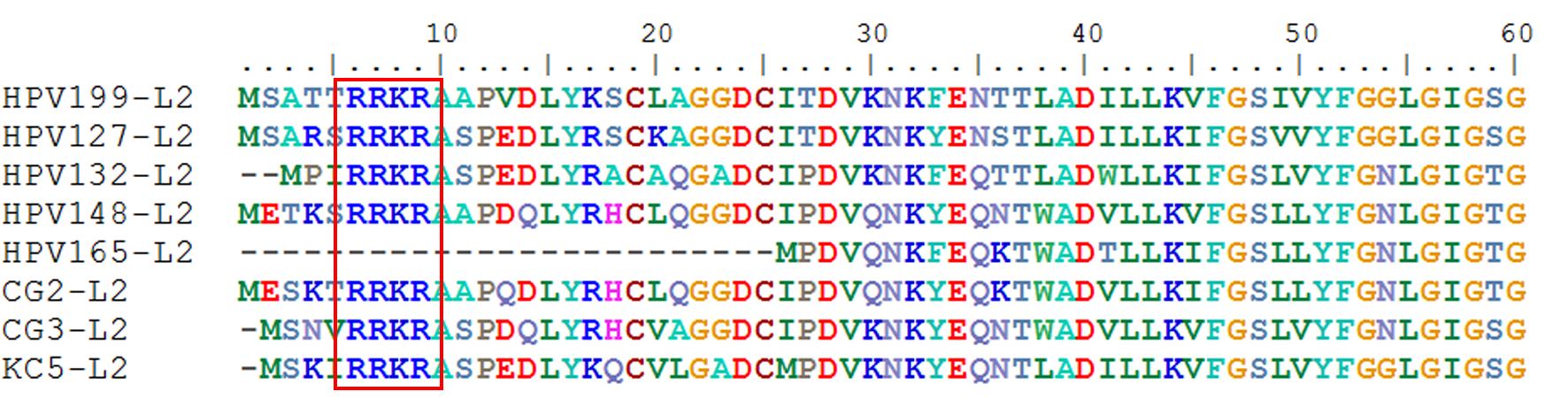

Supplement: S6 Fig — Furin cleavage motifs are indicated with a red box. (TIF) [file pone.0138628.s006.tif]

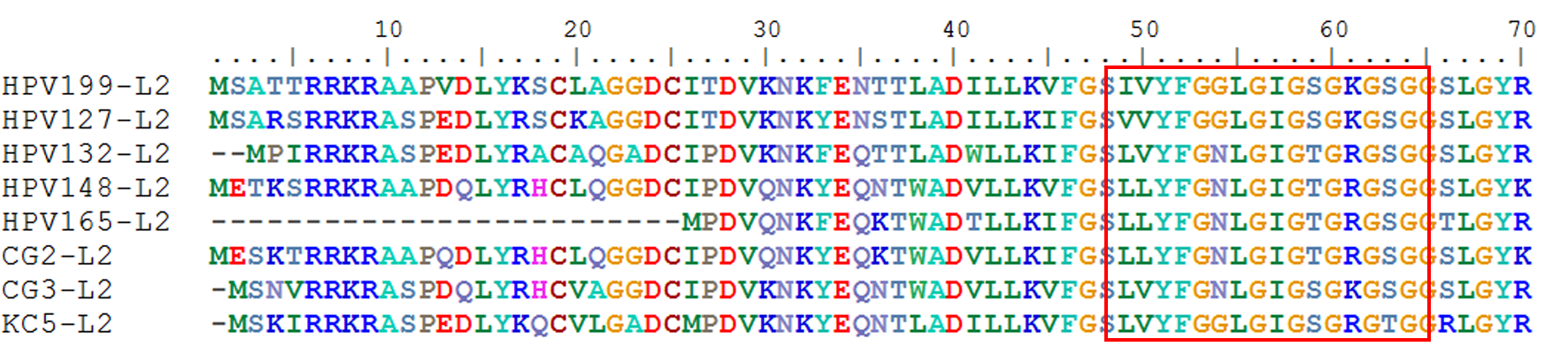

Supplement: S7 Fig — L2 transmembrane domain-like sequences with some minor differences in aa composition are indicated with a red box. (TIF) [file pone.0138628.s007.tif]

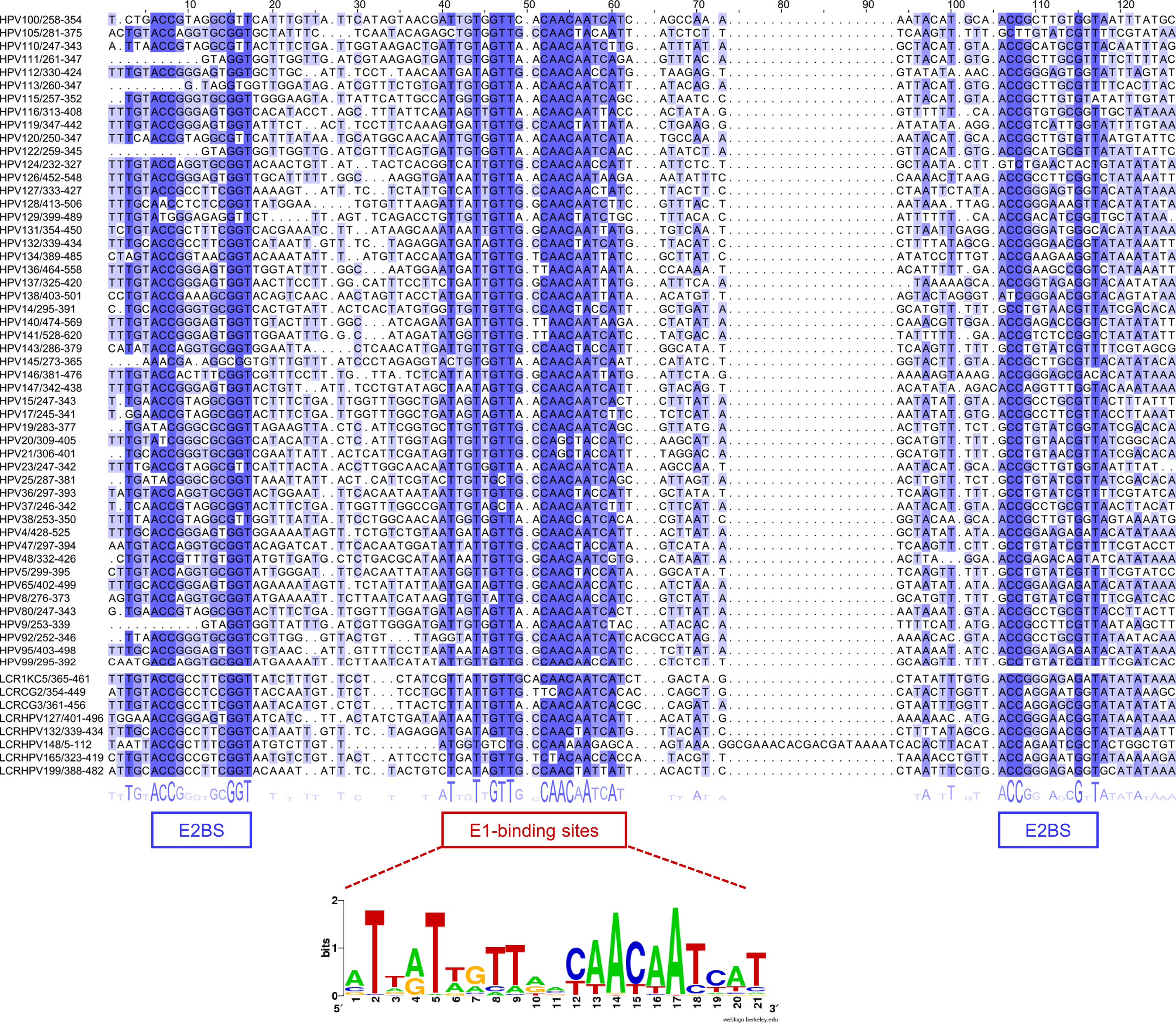

Supplement: S8 Fig — A total of 144 HPV reference sequences was obtained from the Papillomavirus Episteme database (PaVE) and aligned with the Pro-Coffee algorithm of the T-coffee multiple sequence alignment package (http://tcoffee.crg.cat/apps/tcoffee/do:procoffee). The obtained alignment was visualized with the JalView 2.8.1 software package and colored based on the percentage identify option. The E1-binding site sequence logo was derived from the alignment and generated with WebLogo 2.8.2 using the web server at http://weblogo.berkeley.edu/logo.cgi. (TIF) [file pone.0138628.s008.tif]
