# Supplementary material for: Identification of a Novel Human Papillomavirus, Type HPV199, Isolated from a Nasopharynx and Anal Canal, and Complete Genomic Characterization of Papillomavirus Species Gamma-12
Source: PLoS One. 2015 Sep 16;10(9):e0138628. doi: 10.1371/journal.pone.0138628 (PMC4574437; doi:10.1371/journal.pone.0138628)
Supplement: S1 Table — (DOCX) [file pone.0138628.s009.docx]

S1 Table. Primers used for HPV-199 whole genome sequencing using a primer walking strategy

| Primer name | Orientation | Sequence (5'-3') |
| --- | --- | --- |
| KC82-w1f | forward | ACCTCCACCTCAAGGAATAG |
| KC82-w1r | reverse | AGCGTCCTTAGATGATGTAA |
| KC82-w2f | forward | CACCGCCTTCGGTACAAATA |
| KC82-w2r | reverse | GAAGGGGGTTCTTCAATTTC |
| KC82-w3f | forward | ACAGGGTTGATTCTGTTTGT |
| KC82-w3r | reverse | TTCTTCTGGTTGTAGCAGAC |
| KC82-w4f | forward | GCATTTGCAGTTGCCGAAGA |
| KC82-w4r | reverse | TCTGCATCAGGTGCAAACAC |
| KC82-L1f | forward | ACTGCTTATGTAGCCAATACTG |
| KC82-L1r | reverse | AAGTATTAACTAAATCCAGTGAAAC |
