# Supplementary material for: Identification of a Novel Human Papillomavirus, Type HPV199, Isolated from a Nasopharynx and Anal Canal, and Complete Genomic Characterization of Papillomavirus Species Gamma-12
Source: PLoS One. 2015 Sep 16;10(9):e0138628. doi: 10.1371/journal.pone.0138628 (PMC4574437; doi:10.1371/journal.pone.0138628)
Supplement: S2 Table — (DOCX) [file pone.0138628.s010.docx]

S2 Table. Summary of genetic features of the LCR genomic region of current and putative members of species *Gamma*-12

| HPV type | Transcription factors | TATA box (TATAA ) | PolyA site (AATAAA) | E2 binding site(s)  (ACC-N_6-7_-GGT) | E1 binding site |
| --- | --- | --- | --- | --- | --- |
| HPV199 | AP-1, NF-1, Sp1, TFIID, C/EBP | + | + | + | + |
| HPV127 | AP-1, NF-1, Sp1, TFIID | + | + | + | + |
| HPV132 | AP-1, NF-1, Sp1, TFIID | + | + | + | + |
| HPV148 | AP-1, NF-1, Sp1, TFIID | - | + | + | + |
| HPV165 | AP-1, NF-1, Sp1, TFIID, C/EBP | + | + | + | + |
| CG2 | AP-1, NF-1, Sp1, TFIID, C/EBP | + | + | + | + |
| CG3 | AP-1, NF-1, Sp1, TFIID, C/EBP | - | + | + | + |
| CG5 | AP-1, NF-1, Sp1, TFIID, C/EBP | + | + | + | + |
