# Supplementary material for: Identification of a Novel Human Papillomavirus, Type HPV199, Isolated from a Nasopharynx and Anal Canal, and Complete Genomic Characterization of Papillomavirus Species Gamma-12
Source: PLoS One. 2015 Sep 16;10(9):e0138628. doi: 10.1371/journal.pone.0138628 (PMC4574437; doi:10.1371/journal.pone.0138628)
Supplement: S3 Table — (DOCX) [file pone.0138628.s011.docx]

S3 Table. HPV types determined in 916 various clinical specimens using an HPV199 type-specific real-time PCR assay and different primer sets targeting a broad spectrum of HPV types belonging to *Alpha*-PV, *Beta*-PV and *Gamma*-PV

| Sample number | Sample type | HPV199 type-specific RT-PCR | Other HPV types detected |
| --- | --- | --- | --- |
| 1 | common wart tissue | negative | negative^a^ |
| 2 | common wart tissue | negative | negative^a^ |
| 3 | common wart tissue | negative | 57^a^ |
| 4 | common wart tissue | negative | negative^a^ |
| 5 | common wart tissue | negative | negative^a^ |
| 6 | common wart tissue | negative | negative^a^ |
| 7 | common wart tissue | negative | 57^a^ |
| 8 | common wart tissue | negative | negative^a^ |
| 9 | common wart tissue | negative | 125^a^ |
| 10 | common wart tissue | negative | negative^a^ |
| 11 | common wart tissue | negative | negative^a^ |
| 12 | common wart tissue | negative | negative^a^ |
| 13 | common wart tissue | negative | negative^a^ |
| 14 | common wart tissue | negative | negative^a^ |
| 15 | common wart tissue | negative | negative^a^ |
| 16 | common wart tissue | negative | negative^a^ |
| 17 | common wart tissue | negative | negative^a^ |
| 18 | common wart tissue | negative | negative^a^ |
| 19 | common wart tissue | negative | negative^a^ |
| 20 | common wart tissue | negative | negative^a^ |
| 21 | common wart tissue | negative | negative^a^ |
| 22 | common wart tissue | negative | negative^a^ |
| 23 | common wart tissue | negative | negative^a^ |
| 24 | common wart tissue | negative | negative^a^ |
| 25 | common wart tissue | negative | negative^a^ |
| 26 | common wart tissue | negative | negative^a^ |
| 27 | common wart tissue | negative | negative^a^ |
| 28 | common wart tissue | negative | negative^a^ |
| 29 | common wart tissue | negative | negative^a^ |
| 30 | common wart tissue | negative | negative^a^ |
| 31 | common wart tissue | negative | negative^a^ |
| 32 | common wart tissue | negative | negative^a^ |
| 33 | common wart tissue | negative | negative^a^ |
| 34 | common wart tissue | negative | negative^a^ |
| 35 | common wart tissue | negative | negative^a^ |
| 36 | common wart tissue | negative | negative^a^ |
| 37 | common wart tissue | negative | 2^a^ |
| 38 | common wart tissue | negative | negative^a^ |
| 39 | common wart tissue | negative | 57^a^ |
| 40 | common wart tissue | negative | negative^a^ |
| 41 | common wart tissue | negative | 27^a^ |
| 42 | common wart tissue | negative | negative^a^ |
| 43 | common wart tissue | negative | 2^a^ |
| 44 | common wart tissue | negative | negative^a^ |
| 45 | common wart tissue | negative | 57^a^ |
| 46 | common wart tissue | negative | negative^a^ |
| 47 | common wart tissue | negative | 2^a^ |
| 48 | common wart tissue | negative | negative^a^ |
| 49 | common wart tissue | negative | 2^a^ |
| 50 | common wart tissue | negative | negative^a^ |
| 51 | common wart tissue | negative | negative^a^ |
| 52 | common wart tissue | negative | negative^a^ |
| 53 | common wart tissue | negative | negative^a^ |
| 54 | common wart tissue | negative | negative^a^ |
| 55 | common wart tissue | negative | negative^a^ |
| 56 | common wart tissue | negative | negative^a^ |
| 57 | common wart tissue | negative | 57^a^ |
| 58 | common wart tissue | negative | negative^a^ |
| 59 | common wart tissue | negative | 57^b^ |
| 60 | common wart tissue | negative | 57^b^ |
| 61 | common wart tissue | negative | 1, 27^b^ |
| 62 | common wart tissue | negative | 5, 8, 27^b^ |
| 63 | common wart tissue | positive | 2, 27^b^ |
| 64 | common wart tissue | negative | 57^b^ |
| 65 | common wart tissue | negative | 8, 57, 150^b^ |
| 66 | common wart tissue | negative | 57^b^ |
| 67 | common wart tissue | negative | 57^b^ |
| 68 | common wart tissue | negative | negative^b^ |
| 69 | common wart tissue | negative | 57, 105^b^ |
| 70 | common wart tissue | negative | 63^b^ |
| 71 | common wart tissue | negative | 4^b^ |
| 72 | common wart tissue | negative | 2^b^ |
| 73 | common wart tissue | negative | 36, 38^b^ |
| 74 | common wart tissue | negative | 27, 100^b^ |
| 75 | common wart tissue | positive | 2, 5^b^ |
| 76 | common wart tissue | negative | 1, 36^b^ |
| 77 | eyebrow hair follicles | negative | NA |
| 78 | eyebrow hair follicles | negative | NA |
| 79 | eyebrow hair follicles | negative | NA |
| 80 | eyebrow hair follicles | negative | NA |
| 81 | eyebrow hair follicles | negative | NA |
| 82 | eyebrow hair follicles | negative | NA |
| 83 | eyebrow hair follicles | negative | NA |
| 84 | eyebrow hair follicles | negative | NA |
| 85 | eyebrow hair follicles | negative | NA |
| 86 | eyebrow hair follicles | negative | NA |
| 87 | eyebrow hair follicles | negative | NA |
| 88 | eyebrow hair follicles | negative | NA |
| 89 | eyebrow hair follicles | negative | NA |
| 90 | eyebrow hair follicles | negative | NA |
| 91 | eyebrow hair follicles | negative | NA |
| 92 | eyebrow hair follicles | negative | NA |
| 93 | eyebrow hair follicles | negative | NA |
| 94 | eyebrow hair follicles | negative | NA |
| 95 | eyebrow hair follicles | negative | NA |
| 96 | eyebrow hair follicles | negative | NA |
| 97 | eyebrow hair follicles | negative | NA |
| 98 | eyebrow hair follicles | negative | NA |
| 99 | eyebrow hair follicles | negative | NA |
| 100 | eyebrow hair follicles | negative | NA |
| 101 | eyebrow hair follicles | negative | NA |
| 102 | eyebrow hair follicles | negative | NA |
| 103 | eyebrow hair follicles | negative | NA |
| 104 | eyebrow hair follicles | negative | NA |
| 105 | eyebrow hair follicles | negative | NA |
| 106 | eyebrow hair follicles | negative | NA |
| 107 | eyebrow hair follicles | negative | NA |
| 108 | eyebrow hair follicles | negative | NA |
| 109 | eyebrow hair follicles | negative | NA |
| 110 | eyebrow hair follicles | negative | NA |
| 111 | eyebrow hair follicles | negative | NA |
| 112 | eyebrow hair follicles | negative | NA |
| 113 | eyebrow hair follicles | negative | NA |
| 114 | eyebrow hair follicles | negative | NA |
| 115 | eyebrow hair follicles | negative | NA |
| 116 | eyebrow hair follicles | negative | NA |
| 117 | eyebrow hair follicles | negative | NA |
| 118 | eyebrow hair follicles | negative | NA |
| 119 | eyebrow hair follicles | negative | NA |
| 120 | eyebrow hair follicles | negative | NA |
| 121 | eyebrow hair follicles | negative | NA |
| 122 | eyebrow hair follicles | negative | NA |
| 123 | eyebrow hair follicles | negative | NA |
| 124 | eyebrow hair follicles | negative | NA |
| 125 | eyebrow hair follicles | negative | NA |
| 126 | eyebrow hair follicles | negative | NA |
| 127 | eyebrow hair follicles | negative | NA |
| 128 | eyebrow hair follicles | negative | NA |
| 129 | eyebrow hair follicles | negative | NA |
| 130 | eyebrow hair follicles | negative | NA |
| 131 | eyebrow hair follicles | negative | NA |
| 132 | eyebrow hair follicles | negative | NA |
| 133 | eyebrow hair follicles | negative | NA |
| 134 | eyebrow hair follicles | negative | NA |
| 135 | eyebrow hair follicles | negative | NA |
| 136 | eyebrow hair follicles | negative | NA |
| 137 | eyebrow hair follicles | negative | NA |
| 138 | eyebrow hair follicles | negative | NA |
| 139 | eyebrow hair follicles | negative | NA |
| 140 | eyebrow hair follicles | negative | NA |
| 141 | eyebrow hair follicles | negative | NA |
| 142 | eyebrow hair follicles | negative | NA |
| 143 | eyebrow hair follicles | negative | NA |
| 144 | eyebrow hair follicles | negative | NA |
| 145 | eyebrow hair follicles | negative | NA |
| 146 | eyebrow hair follicles | negative | NA |
| 147 | eyebrow hair follicles | negative | NA |
| 148 | eyebrow hair follicles | negative | NA |
| 149 | eyebrow hair follicles | negative | NA |
| 150 | eyebrow hair follicles | negative | NA |
| 151 | eyebrow hair follicles | negative | NA |
| 152 | eyebrow hair follicles | negative | NA |
| 153 | eyebrow hair follicles | negative | NA |
| 154 | eyebrow hair follicles | negative | NA |
| 155 | eyebrow hair follicles | negative | NA |
| 156 | eyebrow hair follicles | negative | NA |
| 157 | eyebrow hair follicles | negative | NA |
| 158 | eyebrow hair follicles | negative | NA |
| 159 | eyebrow hair follicles | negative | NA |
| 160 | eyebrow hair follicles | negative | NA |
| 161 | eyebrow hair follicles | negative | NA |
| 162 | eyebrow hair follicles | negative | NA |
| 163 | eyebrow hair follicles | negative | NA |
| 164 | eyebrow hair follicles | negative | NA |
| 165 | eyebrow hair follicles | negative | NA |
| 166 | eyebrow hair follicles | negative | NA |
| 167 | eyebrow hair follicles | negative | NA |
| 168 | eyebrow hair follicles | negative | NA |
| 169 | eyebrow hair follicles | positive | NA |
| 170 | eyebrow hair follicles | negative | NA |
| 171 | eyebrow hair follicles | negative | NA |
| 172 | eyebrow hair follicles | positive | NA |
| 173 | eyebrow hair follicles | negative | NA |
| 174 | eyebrow hair follicles | negative | NA |
| 175 | eyebrow hair follicles | negative | NA |
| 176 | eyebrow hair follicles | negative | NA |
| 177 | eyebrow hair follicles | negative | NA |
| 178 | eyebrow hair follicles | negative | NA |
| 179 | eyebrow hair follicles | negative | NA |
| 180 | eyebrow hair follicles | negative | NA |
| 181 | eyebrow hair follicles | negative | NA |
| 182 | eyebrow hair follicles | negative | NA |
| 183 | eyebrow hair follicles | negative | NA |
| 184 | eyebrow hair follicles | negative | NA |
| 185 | anal canal swab | negative | 61^c^ |
| 186 | anal canal swab | negative | 53^c^ |
| 187 | anal canal swab | negative | 16^c^ |
| 188 | anal canal swab | negative | negative^c^ |
| 189 | anal canal swab | negative | 11^c^ |
| 190 | anal canal swab | negative | 11, 56^c^ |
| 191 | anal canal swab | negative | negative^c^ |
| 192 | anal canal swab | negative | 16^c^ |
| 193 | anal canal swab | negative | 6^c^ |
| 194 | anal canal swab | negative | 6^c^ |
| 195 | anal canal swab | negative | 52, 83^c^ |
| 196 | anal canal swab | negative | 45, 59, 68^c^ |
| 197 | anal canal swab | negative | 16, 55, 61, 68, 70, 82, 84^c^ |
| 198 | anal canal swab | negative | negative^c^ |
| 199 | anal canal swab | negative | 6, 40, 51, 54, 55, 59, 70, CP6108^c^ |
| 200 | anal canal swab | negative | negative^c^ |
| 201 | anal canal swab | negative | 6, 16, 52, 84, CP6108^c^ |
| 202 | anal canal swab | negative | negative^c^ |
| 203 | anal canal swab | negative | negative^c^ |
| 204 | anal canal swab | negative | 59^c^ |
| 205 | anal canal swab | negative | 6^c^ |
| 206 | anal canal swab | negative | 6, 16, 42, 58, 68, 84, CP6108^c^ |
| 207 | anal canal swab | negative | 16, CP6108^c^ |
| 208 | anal canal swab | negative | negative^c^ |
| 209 | anal canal swab | negative | 51, 70, CP6108^c^ |
| 210 | anal canal swab | positive | negative^c^ |
| 211 | anal canal swab | negative | negative^c^ |
| 212 | anal canal swab | negative | 6^c^ |
| 213 | anal canal swab | negative | 6^c^ |
| 214 | anal canal swab | negative | 16, 18, 68, CP6108^c^ |
| 215 | anal canal swab | negative | 18, 42, 45, 58, 59, 84^c^ |
| 216 | anal canal swab | negative | 18, 31^c^ |
| 217 | anal canal swab | negative | 91^c^ |
| 218 | anal canal swab | negative | 6^c^ |
| 219 | anal canal swab | negative | negative^c^ |
| 220 | anal canal swab | negative | 6, 68, 84, IS39^c^ |
| 221 | anal canal swab | negative | negative^c^ |
| 222 | anal canal swab | negative | 18, 40^c^ |
| 223 | anal canal swab | negative | 35, 45, 52, 53, 82^c^ |
| 224 | anal canal swab | negative | 6, 40, 42, 70, 73^c^ |
| 225 | anal canal swab | negative | 11, 18, 31, 42, 51, 54, 61, 73^c^ |
| 226 | anal canal swab | negative | 73^c^ |
| 227 | anal canal swab | negative | 16, 18, 35, 45, 51, 52, 55, 59, 73, CP6108^c^ |
| 228 | anal canal swab | negative | negative^c^ |
| 229 | anal canal swab | negative | 11, 51, 73^c^ |
| 230 | anal canal swab | negative | negative^c^ |
| 231 | anal canal swab | negative | 31, 58, 73^c^ |
| 232 | anal canal swab | negative | 6, 68^c^ |
| 233 | anal canal swab | negative | 6^c^ |
| 234 | anal canal swab | negative | negative^c^ |
| 235 | anal canal swab | negative | 73^c^ |
| 236 | anal canal swab | negative | 16, 18, 51, 56, 61^c^ |
| 237 | anal canal swab | negative | 91^c^ |
| 238 | anal canal swab | negative | negative^c^ |
| 239 | anal canal swab | negative | 6, 42, 68, CP6108^c^ |
| 240 | anal canal swab | negative | 6^c^ |
| 241 | anal canal swab | negative | 6, 39, 45, 53, 62, 68, 81, 84^c^ |
| 242 | anal canal swab | negative | 6, 16, 40, 45, 51, 56, 81^c^ |
| 243 | anal canal swab | negative | negative^c^ |
| 244 | anal canal swab | negative | 42, 59, 62, 66, IS39, CP6108^c^ |
| 245 | anal canal swab | negative | 18^c^ |
| 246 | anal canal swab | negative | 6, 55, 62, 83^c^ |
| 247 | anal canal swab | negative | 52, 55, 73, CP6108^c^ |
| 248 | anal canal swab | negative | 6, 81, CP6108^c^ |
| 249 | anal canal swab | negative | 6, 11^c^ |
| 250 | anal canal swab | negative | 11, 33, 39, 61, 72^c^ |
| 251 | anal canal swab | negative | 6, 42, 59, 66, 68^c^ |
| 252 | anal canal swab | negative | 6, 16, 18, 52, 68^c^ |
| 253 | anal canal swab | negative | 6, 51^c^ |
| 254 | anal canal swab | negative | 18^c^ |
| 255 | anal canal swab | negative | 42, 62, 67^c^ |
| 256 | anal canal swab | negative | negative^c^ |
| 257 | anal canal swab | negative | 16, 42, 62^c^ |
| 258 | anal canal swab | negative | 11, 42, 45, 66^c^ |
| 259 | anal canal swab | negative | 66^c^ |
| 260 | anal canal swab | negative | negative^c^ |
| 261 | anal canal swab | negative | 6, 53, 54, 83^c^ |
| 262 | anal canal swab | negative | 51^c^ |
| 263 | anal canal swab | negative | 6^c^ |
| 264 | anal canal swab | negative | 6, 84, CP6108^c^ |
| 265 | anal canal swab | negative | 6, 73^c^ |
| 266 | anal canal swab | negative | 6^c^ |
| 267 | anal canal swab | negative | 6, 51, 84^c^ |
| 268 | anal canal swab | negative | negative^c^ |
| 269 | anal canal swab | negative | 6^c^ |
| 270 | anal canal swab | negative | negative^c^ |
| 271 | anal canal swab | negative | negative^c^ |
| 272 | anal canal swab | negative | 6^c^ |
| 273 | anal canal swab | negative | negative^c^ |
| 274 | anal canal swab | negative | 59, CP6108^c^ |
| 275 | anal canal swab | negative | 6, 59^c^ |
| 276 | anal canal swab | negative | negative^c^ |
| 277 | anal canal swab | negative | negative^c^ |
| 278 | anal canal swab | negative | negative^c^ |
| 279 | anal canal swab | negative | 45, 51^c^ |
| 280 | anal canal swab | negative | 16, 52, 54, 58, CP6108^c^ |
| 281 | anal canal swab | negative | 16, 45, 53, 55, 73^c^ |
| 282 | anal canal swab | negative | 53^c^ |
| 283 | anal canal swab | negative | negative^c^ |
| 284 | anal canal swab | negative | 6, 52, 69^c^ |
| 285 | anal canal swab | negative | 52, 55, 56^c^ |
| 286 | anal canal swab | negative | NA |
| 287 | anal canal swab | negative | negative^c^ |
| 288 | anal canal swab | negative | NA |
| 289 | anal canal swab | negative | 31^c^ |
| 290 | anal canal swab | negative | 51^c^ |
| 291 | anal canal swab | negative | 61, 62, 66, 72^c^ |
| 292 | anal canal swab | negative | 6, 70^c^ |
| 293 | anal canal swab | negative | negative^c^ |
| 294 | anal canal swab | negative | NA |
| 295 | anal canal swab | negative | 51^c^ |
| 296 | anal canal swab | negative | 33, 51, CP6108^c^ |
| 297 | anal canal swab | negative | 42, 53^c^ |
| 298 | anal canal swab | negative | NA |
| 299 | anal canal swab | negative | negative^c^ |
| 300 | anal canal swab | negative | 52, 54, 58, CP6108^c^ |
| 301 | anal canal swab | negative | negative^c^ |
| 302 | anal canal swab | negative | 33^c^ |
| 303 | anal canal swab | negative | negative^c^ |
| 304 | anal canal swab | negative | 16, 53, 55, 82^c^ |
| 305 | anal canal swab | negative | 67, CP6108^c^ |
| 306 | anal canal swab | negative | 31, 39, 72^c^ |
| 307 | anal canal swab | negative | 61, 67^c^ |
| 308 | anal canal swab | negative | negative^c^ |
| 309 | anal canal swab | negative | 67, CP6108^c^ |
| 310 | anal canal swab | negative | 11, 31, 33, 53, 58, 59, 61, 70, CP6108^c^ |
| 311 | anal canal swab | negative | 6, 16, 42, 53, 59, 61, CP6108^c^ |
| 312 | anal canal swab | negative | 16, 18, 51, 61^c^ |
| 313 | anal canal swab | negative | 51, 52, 66, 82, 84^c^ |
| 314 | anal canal swab | negative | 51, 68^c^ |
| 315 | anal canal swab | negative | 6, 18, 31, 33, 35, 39, 53, 61^c^ |
| 316 | anal canal swab | negative | 31, 40, 45, 51, 66^c^ |
| 317 | anal canal swab | negative | 59^c^ |
| 318 | anal canal swab | negative | 6, 16, 45, 54, 73^c^ |
| 319 | anal canal swab | negative | 45, 51, 52, 59^c^ |
| 320 | anal canal swab | positive | 31, 45, 52, 59, CP6108^c^ |
| 321 | anal canal swab | negative | 16, 53, 62, 66, 82, 83, 84^c^ |
| 322 | nasopharyngeal swab | negative | 14^d^ |
| 323 | nasopharyngeal swab | negative | X^d^ |
| 324 | nasopharyngeal swab | negative | X^d^ |
| 325 | nasopharyngeal swab | negative | 38^d^ |
| 326 | nasopharyngeal swab | negative | 4^e^ |
| 327 | nasopharyngeal swab | negative | X^d^ |
| 328 | nasopharyngeal swab | negative | negative^d^ |
| 329 | nasopharyngeal swab | negative | negative^d^ |
| 330 | nasopharyngeal swab | negative | 8^d^ |
| 331 | nasopharyngeal swab | negative | negative^d^ |
| 332 | nasopharyngeal swab | negative | 23, 96^d^ |
| 333 | nasopharyngeal swab | negative | 22^d^ |
| 334 | nasopharyngeal swab | negative | 12^d^ |
| 335 | nasopharyngeal swab | negative | negative^d^ |
| 336 | nasopharyngeal swab | negative | negative^d^ |
| 337 | nasopharyngeal swab | negative | 36^d^ |
| 338 | nasopharyngeal swab | negative | 12^d^ |
| 339 | nasopharyngeal swab | negative | negative^d^ |
| 340 | nasopharyngeal swab | negative | X^d^ |
| 341 | nasopharyngeal swab | negative | 9, 38, 93^d^ |
| 342 | nasopharyngeal swab | negative | negative^d^ |
| 343 | nasopharyngeal swab | negative | 9^d^ |
| 344 | nasopharyngeal swab | negative | 5^d^ |
| 345 | nasopharyngeal swab | negative | 4^e^ |
| 346 | nasopharyngeal swab | negative | negative^d^ |
| 347 | nasopharyngeal swab | negative | 9, 80^d^ |
| 348 | nasopharyngeal swab | negative | negative^d^ |
| 349 | nasopharyngeal swab | negative | 4^e^ |
| 350 | nasopharyngeal swab | negative | 15^d^ |
| 351 | nasopharyngeal swab | negative | 12, 15^d^ |
| 352 | nasopharyngeal swab | negative | negative^d^ |
| 353 | nasopharyngeal swab | negative | negative^d^ |
| 354 | nasopharyngeal swab | negative | 93^d^ |
| 355 | nasopharyngeal swab | negative | 4, 12, 49^e^ |
| 356 | nasopharyngeal swab | negative | 4^e^ |
| 357 | nasopharyngeal swab | negative | 37^d^ |
| 358 | nasopharyngeal swab | negative | negative^d^ |
| 359 | nasopharyngeal swab | negative | X^d^ |
| 360 | nasopharyngeal swab | negative | 9^d^ |
| 361 | nasopharyngeal swab | negative | 8^d^ |
| 362 | nasopharyngeal swab | negative | negative^d^ |
| 363 | nasopharyngeal swab | negative | 37^d^ |
| 364 | nasopharyngeal swab | negative | 20^d^ |
| 365 | nasopharyngeal swab | negative | 5, 8, 9, 14, 17, 19, 20, 21, 80^d^ |
| 366 | nasopharyngeal swab | negative | 22^d^ |
| 367 | nasopharyngeal swab | negative | 93^d^ |
| 368 | nasopharyngeal swab | negative | 22^d^ |
| 369 | nasopharyngeal swab | negative | 38, 80^d, e^ |
| 370 | nasopharyngeal swab | negative | negative^d^ |
| 371 | nasopharyngeal swab | negative | 8, 23, 36, 37^d^ |
| 372 | nasopharyngeal swab | negative | 21, 24, 75^d^ |
| 373 | nasopharyngeal swab | negative | X^d^ |
| 374 | nasopharyngeal swab | negative | 23^d^ |
| 375 | nasopharyngeal swab | negative | 9^d^ |
| 376 | nasopharyngeal swab | negative | negative^d^ |
| 377 | nasopharyngeal swab | negative | 12, 93^d^ |
| 378 | nasopharyngeal swab | negative | 4^e^ |
| 379 | nasopharyngeal swab | negative | 20^d^ |
| 380 | nasopharyngeal swab | negative | negative^d^ |
| 381 | nasopharyngeal swab | negative | negative^d^ |
| 382 | nasopharyngeal swab | negative | 12^d^ |
| 383 | nasopharyngeal swab | negative | 9^d^ |
| 384 | nasopharyngeal swab | negative | 19^d^ |
| 385 | nasopharyngeal swab | negative | X^d^ |
| 386 | nasopharyngeal swab | negative | 47^d^ |
| 387 | nasopharyngeal swab | negative | negative^d^ |
| 388 | nasopharyngeal swab | negative | 38^d^ |
| 389 | nasopharyngeal swab | negative | 8^d^ |
| 390 | nasopharyngeal swab | negative | 92^d^ |
| 391 | nasopharyngeal swab | negative | negative^d^ |
| 392 | nasopharyngeal swab | negative | 80^d^ |
| 393 | nasopharyngeal swab | negative | 8^d^ |
| 394 | nasopharyngeal swab | negative | negative^d^ |
| 395 | nasopharyngeal swab | negative | 5^d^ |
| 396 | nasopharyngeal swab | negative | negative^d^ |
| 397 | nasopharyngeal swab | positive | 17^d^ |
| 398 | nasopharyngeal swab | negative | 19^d^ |
| 399 | nasopharyngeal swab | negative | 36, 38^d^ |
| 400 | nasopharyngeal swab | negative | 19^d^ |
| 401 | nasopharyngeal swab | negative | 25^e^ |
| 402 | nasopharyngeal swab | negative | 23, 38^d^ |
| 403 | nasopharyngeal swab | negative | 4^e^ |
| 404 | nasopharyngeal swab | negative | 4, 20^e^ |
| 405 | nasopharyngeal swab | negative | 38^d^ |
| 406 | nasopharyngeal swab | negative | X^d^ |
| 407 | nasopharyngeal swab | negative | 49^d^ |
| 408 | nasopharyngeal swab | negative | 9^d^ |
| 409 | nasopharyngeal swab | negative | 15^d^ |
| 410 | nasopharyngeal swab | negative | 20^d^ |
| 411 | nasopharyngeal swab | negative | negative^e^ |
| 412 | nasopharyngeal swab | negative | negative^d^ |
| 413 | nasopharyngeal swab | negative | negative^d^ |
| 414 | nasopharyngeal swab | negative | 8, 17, 23, 80^d^ |
| 415 | nasopharyngeal swab | negative | negative^d^ |
| 416 | nasopharyngeal swab | negative | 38^d^ |
| 417 | nasopharyngeal swab | negative | 9, 36, 92, 96^d^ |
| 418 | nasopharyngeal swab | negative | 23^d^ |
| 419 | nasopharyngeal swab | positive | negative^d^ |
| 420 | nasopharyngeal swab | negative | negative^d^ |
| 421 | nasopharyngeal swab | negative | 38^d^ |
| 422 | nasopharyngeal swab | negative | negative^d^ |
| 423 | nasopharyngeal swab | positive | 22, 49^d^ |
| 424 | nasopharyngeal swab | negative | 20^d^ |
| 425 | nasopharyngeal swab | negative | 5^d^ |
| 426 | nasopharyngeal swab | negative | 9, 20^d^ |
| 427 | nasopharyngeal swab | negative | X^d^ |
| 428 | nasopharyngeal swab | negative | negative^d^ |
| 429 | nasopharyngeal swab | negative | 38^d^ |
| 430 | nasopharyngeal swab | negative | 19^d^ |
| 431 | nasopharyngeal swab | negative | 15, 37, 80^d^ |
| 432 | nasopharyngeal swab | negative | 22^d^ |
| 433 | nasopharyngeal swab | negative | 4^e^ |
| 434 | nasopharyngeal swab | negative | 23, 24^d^ |
| 435 | nasopharyngeal swab | negative | 38^d^ |
| 436 | nasopharyngeal swab | negative | 12, 23, 24^d^ |
| 437 | nasopharyngeal swab | negative | 15, 23, 38^d^ |
| 438 | nasopharyngeal swab | negative | 17, 38, 49^d^ |
| 439 | nasopharyngeal swab | negative | negative^d^ |
| 440 | nasopharyngeal swab | negative | negative^d^ |
| 441 | nasopharyngeal swab | negative | X^d^ |
| 442 | nasopharyngeal swab | negative | X^d^ |
| 443 | nasopharyngeal swab | negative | 17, 37^d^ |
| 444 | nasopharyngeal swab | negative | X^d^ |
| 445 | nasopharyngeal swab | negative | 93^d^ |
| 446 | nasopharyngeal swab | negative | 38^d^ |
| 447 | nasopharyngeal swab | negative | 38^d^ |
| 448 | nasopharyngeal swab | negative | 23^d^ |
| 449 | nasopharyngeal swab | negative | 17, 80^d^ |
| 450 | nasopharyngeal swab | negative | 8, 9, 24, 93^d^ |
| 451 | nasopharyngeal swab | negative | 37^d^ |
| 452 | nasopharyngeal swab | negative | negative^d^ |
| 453 | nasopharyngeal swab | negative | negative^e^ |
| 454 | nasopharyngeal swab | negative | negative^d^ |
| 455 | nasopharyngeal swab | negative | 36^d^ |
| 456 | nasopharyngeal swab | negative | 23^d^ |
| 457 | nasopharyngeal swab | negative | 38^d^ |
| 458 | nasopharyngeal swab | negative | 8, 93^d^ |
| 459 | nasopharyngeal swab | negative | 23^d^ |
| 460 | nasopharyngeal swab | negative | 8^d^ |
| 461 | nasopharyngeal swab | negative | negative^d^ |
| 462 | nasopharyngeal swab | negative | 8, 20, 21, 38^d^ |
| 463 | nasopharyngeal swab | negative | negative^d^ |
| 464 | nasopharyngeal swab | negative | 17, 23, 75^d^ |
| 465 | nasopharyngeal swab | negative | 17^d^ |
| 466 | nasopharyngeal swab | negative | negative^d^ |
| 467 | nasopharyngeal swab | negative | X^d^ |
| 468 | nasopharyngeal swab | negative | negative^d^ |
| 469 | nasopharyngeal swab | negative | 38, 49^d^ |
| 470 | nasopharyngeal swab | negative | 12,23^d^ |
| 471 | nasopharyngeal swab | negative | 17, 23, 93^d^ |
| 472 | nasopharyngeal swab | negative | negative^d^ |
| 473 | nasopharyngeal swab | negative | negative^d^ |
| 474 | nasopharyngeal swab | negative | negative^d^ |
| 475 | nasopharyngeal swab | negative | 8, 22^d^ |
| 476 | nasopharyngeal swab | negative | negative^d^ |
| 477 | nasopharyngeal swab | negative | negative^d^ |
| 478 | nasopharyngeal swab | negative | negative^d^ |
| 479 | nasopharyngeal swab | negative | negative^d^ |
| 480 | nasopharyngeal swab | negative | 36^d^ |
| 481 | nasopharyngeal swab | negative | negative^d^ |
| 482 | nasopharyngeal swab | negative | 76^d^ |
| 483 | nasopharyngeal swab | negative | 37^d^ |
| 484 | nasopharyngeal swab | negative | 8, 23^d^ |
| 485 | nasopharyngeal swab | negative | 15^d^ |
| 486 | nasopharyngeal swab | negative | 80^d^ |
| 487 | nasopharyngeal swab | negative | X^d^ |
| 488 | nasopharyngeal swab | positive | 12^d^ |
| 489 | nasopharyngeal swab | negative | 5, 19 |
| 490 | nasopharyngeal swab | negative | 17^d^ |
| 491 | nasopharyngeal swab | negative | 4, 15, 96^d, e^ |
| 492 | nasopharyngeal swab | negative | negative^d^ |
| 493 | nasopharyngeal swab | negative | X^d^ |
| 494 | nasopharyngeal swab | negative | 38^d^ |
| 495 | nasopharyngeal swab | negative | X^d^ |
| 496 | nasopharyngeal swab | negative | 17, 23, 96^d^ |
| 497 | nasopharyngeal swab | negative | negative^d^ |
| 498 | nasopharyngeal swab | negative | negative^d^ |
| 499 | nasopharyngeal swab | negative | 5^d^ |
| 500 | nasopharyngeal swab | negative | negative^d^ |
| 501 | nasopharyngeal swab | negative | 23, 38^d^ |
| 502 | nasopharyngeal swab | negative | 19, 25, 36, 92^d^ |
| 503 | nasopharyngeal swab | negative | negative^d^ |
| 504 | nasopharyngeal swab | negative | 15, 20, 38^d^ |
| 505 | nasopharyngeal swab | negative | negative^d^ |
| 506 | cervical swab | negative | negative^c^ |
| 507 | cervical swab | negative | negative^c^ |
| 508 | cervical swab | negative | negative^c^ |
| 509 | cervical swab | negative | negative^c^ |
| 510 | cervical swab | negative | 54, 89^c^ |
| 511 | cervical swab | negative | negative^c^ |
| 512 | cervical swab | negative | negative^c^ |
| 513 | cervical swab | negative | negative^c^ |
| 514 | cervical swab | negative | negative^c^ |
| 515 | cervical swab | negative | negative^c^ |
| 516 | cervical swab | negative | negative^c^ |
| 517 | cervical swab | negative | negative^c^ |
| 518 | cervical swab | negative | negative^c^ |
| 519 | cervical swab | negative | negative^c^ |
| 520 | cervical swab | negative | 70^c^ |
| 521 | cervical swab | negative | negative^c^ |
| 522 | cervical swab | negative | negative^c^ |
| 523 | cervical swab | negative | negative^c^ |
| 524 | cervical swab | negative | negative^c^ |
| 525 | cervical swab | negative | negative^c^ |
| 526 | cervical swab | negative | negative^c^ |
| 527 | cervical swab | negative | negative^c^ |
| 528 | cervical swab | negative | negative^c^ |
| 529 | cervical swab | negative | negative^c^ |
| 530 | cervical swab | negative | negative^c^ |
| 531 | cervical swab | negative | negative^c^ |
| 532 | cervical swab | negative | negative^c^ |
| 533 | cervical swab | negative | negative^c^ |
| 534 | cervical swab | negative | negative^c^ |
| 535 | cervical swab | negative | negative^c^ |
| 536 | cervical swab | negative | negative^c^ |
| 537 | cervical swab | negative | negative^c^ |
| 538 | cervical swab | negative | negative^c^ |
| 539 | cervical swab | negative | negative^c^ |
| 540 | cervical swab | negative | negative^c^ |
| 541 | cervical swab | negative | negative^c^ |
| 542 | cervical swab | negative | negative^c^ |
| 543 | cervical swab | negative | negative^c^ |
| 544 | cervical swab | negative | negative^c^ |
| 545 | cervical swab | negative | negative^c^ |
| 546 | cervical swab | negative | negative^c^ |
| 547 | cervical swab | negative | negative^c^ |
| 548 | cervical swab | negative | negative^c^ |
| 549 | cervical swab | negative | negative^c^ |
| 550 | cervical swab | negative | negative^c^ |
| 551 | cervical swab | negative | negative^c^ |
| 552 | cervical swab | negative | negative^c^ |
| 553 | cervical swab | negative | negative^c^ |
| 554 | cervical swab | negative | negative^c^ |
| 555 | cervical swab | negative | negative^c^ |
| 556 | cervical swab | negative | negative^c^ |
| 557 | cervical swab | negative | negative^c^ |
| 558 | cervical swab | negative | negative^c^ |
| 559 | cervical swab | negative | negative^c^ |
| 560 | cervical swab | negative | negative^c^ |
| 561 | cervical swab | negative | negative^c^ |
| 562 | cervical swab | negative | negative^c^ |
| 563 | cervical swab | negative | negative^c^ |
| 564 | cervical swab | negative | negative^c^ |
| 565 | cervical swab | negative | negative^c^ |
| 566 | cervical swab | negative | negative^c^ |
| 567 | cervical swab | negative | negative^c^ |
| 568 | cervical swab | negative | negative^c^ |
| 569 | cervical swab | negative | negative^c^ |
| 570 | cervical swab | negative | negative^c^ |
| 571 | cervical swab | negative | 9, 16, 31, 33^c^ |
| 572 | cervical swab | negative | negative^c^ |
| 573 | cervical swab | negative | negative^c^ |
| 574 | cervical swab | negative | negative^c^ |
| 575 | cervical swab | negative | negative^c^ |
| 576 | cervical swab | negative | negative^c^ |
| 577 | cervical swab | negative | negative^c^ |
| 578 | cervical swab | negative | negative^c^ |
| 579 | cervical swab | negative | negative^c^ |
| 580 | cervical swab | negative | negative^c^ |
| 581 | cervical swab | negative | 59^c^ |
| 582 | cervical swab | negative | negative^c^ |
| 583 | cervical swab | negative | negative^c^ |
| 584 | cervical swab | negative | negative^c^ |
| 585 | cervical swab | negative | negative^c^ |
| 586 | cervical swab | negative | negative^c^ |
| 587 | cervical swab | negative | negative^c^ |
| 588 | cervical swab | negative | 15^c^ |
| 589 | cervical swab | negative | negative^c^ |
| 590 | cervical swab | negative | negative^c^ |
| 591 | cervical swab | negative | negative^c^ |
| 592 | cervical swab | negative | negative^c^ |
| 593 | cervical swab | negative | negative^c^ |
| 594 | cervical swab | negative | negative^c^ |
| 595 | cervical swab | negative | negative^c^ |
| 596 | cervical swab | negative | negative^c^ |
| 597 | cervical swab | negative | negative^c^ |
| 598 | cervical swab | negative | negative^c^ |
| 599 | cervical swab | negative | negative^c^ |
| 600 | cervical swab | negative | negative^c^ |
| 601 | cervical swab | negative | negative^c^ |
| 602 | cervical swab | negative | negative^c^ |
| 603 | cervical swab | negative | negative^c^ |
| 604 | cervical swab | negative | negative^c^ |
| 605 | cervical swab | negative | negative^c^ |
| 606 | cervical swab | negative | negative^c^ |
| 607 | cervical swab | negative | negative^c^ |
| 608 | cervical swab | negative | negative^c^ |
| 609 | cervical swab | negative | negative^c^ |
| 610 | cervical swab | negative | negative^c^ |
| 611 | cervical swab | positive | negative^c^ |
| 612 | cervical swab | negative | negative^c^ |
| 613 | cervical swab | positive | negative^c^ |
| 614 | cervical swab | negative | negative^c^ |
| 615 | cervical swab | negative | negative^c^ |
| 616 | cervical swab | negative | negative^c^ |
| 617 | cervical swab | negative | negative^c^ |
| 618 | cervical swab | negative | negative^c^ |
| 619 | cervical swab | negative | negative^c^ |
| 620 | cervical swab | negative | negative^c^ |
| 621 | cervical swab | negative | negative^c^ |
| 622 | cervical swab | negative | negative^c^ |
| 623 | cervical swab | negative | negative^c^ |
| 624 | cervical swab | negative | negative^c^ |
| 625 | cervical swab | negative | negative^c^ |
| 626 | cervical swab | negative | negative^c^ |
| 627 | cervical swab | negative | negative^c^ |
| 628 | cervical swab | negative | negative^c^ |
| 629 | cervical swab | negative | negative^c^ |
| 630 | cervical swab | negative | negative^c^ |
| 631 | cervical swab | negative | 73, 83, 89^c^ |
| 632 | cervical swab | negative | negative^c^ |
| 633 | cervical swab | negative | negative^c^ |
| 634 | cervical swab | negative | negative^c^ |
| 635 | cervical swab | negative | negative^c^ |
| 636 | cervical swab | negative | negative^c^ |
| 637 | cervical swab | negative | negative^c^ |
| 638 | cervical swab | negative | negative^c^ |
| 639 | cervical swab | negative | negative^c^ |
| 640 | cervical swab | negative | negative^c^ |
| 641 | cervical swab | negative | negative^c^ |
| 642 | cervical swab | negative | negative^c^ |
| 643 | cervical swab | negative | negative^c^ |
| 644 | cervical swab | negative | negative^c^ |
| 645 | cervical swab | negative | 42, 54^c^ |
| 646 | cervical swab | negative | negative^c^ |
| 647 | cervical swab | negative | negative^c^ |
| 648 | cervical swab | negative | negative^c^ |
| 649 | cervical swab | negative | negative^c^ |
| 650 | cervical swab | negative | negative^c^ |
| 651 | cervical swab | negative | negative^c^ |
| 652 | cervical swab | negative | negative^c^ |
| 653 | cervical swab | negative | negative^c^ |
| 654 | cervical swab | negative | negative^c^ |
| 655 | cervical swab | negative | negative^c^ |
| 656 | cervical swab | negative | negative^c^ |
| 657 | cervical swab | negative | negative^c^ |
| 658 | cervical swab | negative | negative^c^ |
| 659 | cervical swab | negative | negative^c^ |
| 660 | cervical swab | negative | negative^c^ |
| 661 | cervical swab | negative | negative^c^ |
| 662 | cervical swab | negative | negative^c^ |
| 663 | cervical swab | negative | negative^c^ |
| 664 | cervical swab | negative | negative^c^ |
| 665 | cervical swab | negative | negative^c^ |
| 666 | cervical swab | negative | negative^c^ |
| 667 | cervical swab | negative | negative^c^ |
| 668 | cervical swab | negative | negative^c^ |
| 669 | cervical swab | negative | negative^c^ |
| 670 | cervical swab | negative | negative^c^ |
| 671 | cervical swab | negative | negative^c^ |
| 672 | cervical swab | negative | negative^c^ |
| 673 | cervical swab | negative | negative^c^ |
| 674 | cervical swab | negative | negative^c^ |
| 675 | cervical swab | negative | negative^c^ |
| 676 | cervical swab | negative | negative^c^ |
| 677 | cervical swab | negative | negative^c^ |
| 678 | cervical swab | negative | negative^c^ |
| 679 | cervical swab | negative | negative^c^ |
| 680 | cervical swab | negative | negative^c^ |
| 681 | cervical swab | negative | negative^c^ |
| 682 | cervical swab | negative | negative^c^ |
| 683 | cervical swab | negative | negative^c^ |
| 684 | cervical swab | negative | negative^c^ |
| 685 | cervical swab | negative | negative^c^ |
| 686 | cervical swab | negative | negative^c^ |
| 687 | cervical swab | negative | negative^c^ |
| 688 | cervical swab | negative | negative^c^ |
| 689 | cervical swab | negative | negative^c^ |
| 690 | cervical swab | negative | negative^c^ |
| 691 | cervical swab | negative | negative^c^ |
| 692 | cervical swab | negative | negative^c^ |
| 693 | cervical swab | negative | negative^c^ |
| 694 | cervical swab | negative | negative^c^ |
| 695 | cervical swab | negative | negative^c^ |
| 696 | cervical swab | negative | negative^c^ |
| 697 | cervical swab | negative | negative^c^ |
| 698 | cervical swab | negative | negative^c^ |
| 699 | cervical swab | negative | negative^c^ |
| 700 | cervical swab | negative | negative^c^ |
| 701 | cervical swab | negative | negative^c^ |
| 702 | cervical swab | negative | negative^c^ |
| 703 | cervical swab | negative | negative^c^ |
| 704 | cervical swab | negative | negative^c^ |
| 705 | cervical swab | negative | negative^c^ |
| 706 | cervical swab | negative | negative^c^ |
| 707 | cervical swab | negative | negative^c^ |
| 708 | cervical swab | negative | negative^c^ |
| 709 | cervical swab | negative | negative^c^ |
| 710 | cervical swab | negative | negative^c^ |
| 711 | cervical swab | negative | negative^c^ |
| 712 | cervical swab | negative | negative^c^ |
| 713 | cervical swab | negative | negative^c^ |
| 714 | cervical swab | negative | negative^c^ |
| 715 | cervical swab | negative | negative^c^ |
| 716 | cervical swab | negative | negative^c^ |
| 717 | cervical swab | negative | negative^c^ |
| 718 | cervical swab | negative | negative^c^ |
| 719 | cervical swab | negative | negative^c^ |
| 720 | cervical swab | negative | negative^c^ |
| 721 | cervical swab | negative | negative^c^ |
| 722 | cervical swab | negative | negative^c^ |
| 723 | cervical swab | negative | 62^c^ |
| 724 | cervical swab | negative | negative^c^ |
| 725 | cervical swab | negative | negative^c^ |
| 726 | cervical swab | negative | negative^c^ |
| 727 | cervical swab | negative | negative^c^ |
| 728 | cervical swab | negative | negative^c^ |
| 729 | cervical swab | negative | negative^c^ |
| 730 | cervical swab | negative | negative^c^ |
| 731 | cervical swab | negative | negative^c^ |
| 732 | cervical swab | negative | negative^c^ |
| 733 | cervical swab | negative | negative^c^ |
| 734 | cervical swab | negative | negative^c^ |
| 735 | cervical swab | negative | negative^c^ |
| 736 | cervical swab | negative | negative^c^ |
| 737 | cervical swab | negative | negative^c^ |
| 738 | cervical swab | negative | negative^c^ |
| 739 | cervical swab | negative | negative^c^ |
| 740 | cervical swab | negative | negative^c^ |
| 741 | cervical swab | negative | negative^c^ |
| 742 | cervical swab | negative | negative^c^ |
| 743 | cervical swab | negative | negative^c^ |
| 744 | cervical swab | negative | negative^c^ |
| 745 | cervical swab | negative | negative^c^ |
| 746 | cervical swab | negative | negative^c^ |
| 747 | cervical swab | negative | negative^c^ |
| 748 | cervical swab | negative | negative^c^ |
| 749 | cervical swab | negative | negative^c^ |
| 750 | cervical swab | negative | negative^c^ |
| 751 | cervical swab | negative | negative^c^ |
| 752 | cervical swab | negative | negative^c^ |
| 753 | cervical swab | negative | negative^c^ |
| 754 | cervical swab | negative | negative^c^ |
| 755 | cervical swab | negative | negative^c^ |
| 756 | cervical swab | negative | negative^c^ |
| 757 | cervical swab | negative | negative^c^ |
| 758 | cervical swab | negative | negative^c^ |
| 759 | cervical swab | negative | negative^c^ |
| 760 | cervical swab | negative | negative^c^ |
| 761 | cervical swab | negative | negative^c^ |
| 762 | cervical swab | negative | negative^c^ |
| 763 | cervical swab | negative | negative^c^ |
| 764 | cervical swab | negative | negative^c^ |
| 765 | cervical swab | negative | negative^c^ |
| 766 | cervical swab | negative | negative^c^ |
| 767 | cervical swab | negative | negative^c^ |
| 768 | cervical swab | negative | negative^c^ |
| 769 | cervical swab | negative | negative^c^ |
| 770 | cervical swab | negative | negative^c^ |
| 771 | cervical swab | negative | negative^c^ |
| 772 | cervical swab | negative | negative^c^ |
| 773 | cervical swab | negative | negative^c^ |
| 774 | cervical swab | negative | negative^c^ |
| 775 | cervical swab | negative | negative^c^ |
| 776 | cervical swab | negative | negative^c^ |
| 777 | cervical swab | negative | negative^c^ |
| 778 | cervical swab | negative | negative^c^ |
| 779 | cervical swab | negative | negative^c^ |
| 780 | cervical swab | negative | negative^c^ |
| 781 | cervical swab | negative | negative^c^ |
| 782 | cervical swab | negative | negative^c^ |
| 783 | cervical swab | negative | negative^c^ |
| 784 | cervical swab | negative | negative^c^ |
| 785 | cervical swab | negative | negative^c^ |
| 786 | cervical swab | negative | negative^c^ |
| 787 | cervical swab | negative | negative^c^ |
| 788 | cervical swab | negative | negative^c^ |
| 789 | cervical swab | negative | negative^c^ |
| 790 | cervical swab | negative | negative^c^ |
| 791 | cervical swab | negative | negative^c^ |
| 792 | cervical swab | negative | negative^c^ |
| 793 | cervical swab | negative | negative^c^ |
| 794 | cervical swab | negative | 44^c^ |
| 795 | cervical swab | negative | negative^c^ |
| 796 | cervical swab | negative | negative^c^ |
| 797 | cervical swab | negative | negative^c^ |
| 798 | cervical swab | negative | negative^c^ |
| 799 | cervical swab | negative | negative^c^ |
| 800 | cervical swab | negative | negative^c^ |
| 801 | cervical swab | negative | negative^c^ |
| 802 | cervical swab | negative | negative^c^ |
| 803 | cervical swab | negative | negative^c^ |
| 804 | cervical swab | negative | negative^c^ |
| 805 | cervical swab | negative | negative^c^ |
| 806 | cervical swab | negative | negative^c^ |
| 807 | cervical swab | negative | negative^c^ |
| 808 | cervical swab | negative | negative^c^ |
| 809 | cervical swab | negative | negative^c^ |
| 810 | cervical swab | negative | negative^c^ |
| 811 | cervical swab | negative | negative^c^ |
| 812 | cervical swab | negative | negative^c^ |
| 813 | cervical swab | negative | negative^c^ |
| 814 | cervical swab | negative | negative^c^ |
| 815 | cervical swab | negative | negative^c^ |
| 816 | cervical swab | negative | negative^c^ |
| 817 | cervical swab | negative | negative^c^ |
| 818 | cervical swab | negative | negative^c^ |
| 819 | cervical swab | negative | negative^c^ |
| 820 | cervical swab | negative | negative^c^ |
| 821 | cervical swab | negative | negative^c^ |
| 822 | cervical swab | negative | negative^c^ |
| 823 | cervical swab | negative | negative^c^ |
| 824 | cervical swab | negative | negative^c^ |
| 825 | cervical swab | negative | negative^c^ |
| 826 | cervical swab | negative | negative^c^ |
| 827 | cervical swab | negative | negative^c^ |
| 828 | cervical swab | negative | negative^c^ |
| 829 | cervical swab | negative | negative^c^ |
| 830 | cervical swab | negative | negative^c^ |
| 831 | cervical swab | negative | negative^c^ |
| 832 | cervical swab | negative | negative^c^ |
| 833 | cervical swab | negative | negative^c^ |
| 834 | cervical swab | negative | negative^c^ |
| 835 | cervical swab | negative | negative^c^ |
| 836 | cervical swab | negative | negative^c^ |
| 837 | cervical swab | negative | negative^c^ |
| 838 | cervical swab | negative | negative^c^ |
| 839 | cervical swab | negative | negative^c^ |
| 840 | cervical swab | negative | negative^c^ |
| 841 | cervical swab | negative | negative^c^ |
| 842 | cervical swab | negative | negative^c^ |
| 843 | cervical swab | negative | negative^c^ |
| 844 | cervical swab | negative | negative^c^ |
| 845 | cervical swab | negative | negative^c^ |
| 846 | cervical swab | negative | negative^c^ |
| 847 | cervical swab | negative | negative^c^ |
| 848 | cervical swab | negative | negative^c^ |
| 849 | cervical swab | negative | negative^c^ |
| 850 | cervical swab | negative | negative^c^ |
| 851 | cervical swab | negative | negative^c^ |
| 852 | cervical swab | negative | 61^c^ |
| 853 | cervical swab | negative | negative^c^ |
| 854 | cervical swab | negative | negative^c^ |
| 855 | cervical swab | negative | negative^c^ |
| 856 | cervical swab | negative | negative^c^ |
| 857 | cervical swab | negative | negative^c^ |
| 858 | cervical swab | negative | negative^c^ |
| 859 | cervical swab | negative | negative^c^ |
| 860 | cervical swab | negative | negative^c^ |
| 861 | cervical swab | negative | negative^c^ |
| 862 | cervical swab | negative | negative^c^ |
| 863 | cervical swab | negative | negative^c^ |
| 864 | cervical swab | negative | negative^c^ |
| 865 | cervical swab | negative | negative^c^ |
| 866 | cervical swab | negative | negative^c^ |
| 867 | cervical swab | negative | negative^c^ |
| 868 | cervical swab | negative | negative^c^ |
| 869 | cervical swab | negative | negative^c^ |
| 870 | cervical swab | negative | negative^c^ |
| 871 | cervical swab | negative | 6, 53^c^ |
| 872 | cervical swab | negative | negative^c^ |
| 873 | cervical swab | negative | negative^c^ |
| 874 | cervical swab | negative | negative^c^ |
| 875 | cervical swab | negative | negative^c^ |
| 876 | cervical swab | negative | negative^c^ |
| 877 | cervical swab | negative | negative^c^ |
| 878 | cervical swab | negative | negative^c^ |
| 879 | cervical swab | negative | negative^c^ |
| 880 | cervical swab | negative | negative^c^ |
| 881 | cervical swab | negative | negative^c^ |
| 882 | cervical swab | negative | negative^c^ |
| 883 | cervical swab | negative | negative^c^ |
| 884 | cervical swab | negative | 53^c^ |
| 885 | cervical swab | negative | negative^c^ |
| 886 | cervical swab | negative | negative^c^ |
| 887 | cervical swab | negative | negative^c^ |
| 888 | cervical swab | negative | negative^c^ |
| 889 | cervical swab | negative | negative^c^ |
| 890 | cervical swab | negative | negative^c^ |
| 891 | cervical swab | negative | negative^c^ |
| 892 | cervical swab | positive | negative^c^ |
| 893 | cervical swab | negative | negative^c^ |
| 894 | cervical swab | negative | negative^c^ |
| 895 | cervical swab | negative | negative^c^ |
| 896 | cervical swab | negative | negative^c^ |
| 897 | cervical swab | negative | negative^c^ |
| 898 | cervical swab | negative | negative^c^ |
| 899 | cervical swab | negative | negative^c^ |
| 900 | cervical swab | negative | negative^c^ |
| 901 | cervical swab | negative | negative^c^ |
| 902 | cervical swab | negative | negative^c^ |
| 903 | cervical swab | negative | negative^c^ |
| 904 | cervical swab | negative | negative^c^ |
| 905 | cervical swab | negative | negative^c^ |
| 906 | cervical swab | negative | negative^c^ |
| 907 | cervical swab | negative | negative^c^ |
| 908 | cervical swab | negative | negative^c^ |
| 909 | cervical swab | negative | negative^c^ |
| 910 | cervical swab | negative | negative^c^ |
| 911 | cervical swab | negative | negative^c^ |
| 912 | cervical swab | negative | negative^c^ |
| 913 | cervical swab | negative | negative^c^ |
| 914 | cervical swab | negative | negative^c^ |
| 915 | cervical swab | negative | negative^c^ |
| 916 | cervical swab | negative | negative^c^ |

^a^Clinical specimens were tested using in-house test with primer sets targeting the following HPV genotypes belonging to *Alpha*-PV: HPV2, HPV3, HPV6, HPV7, HPV10, HPV11, HPV13, HPV27, HPV28, HPV29, HPV32, HPV40, HPV42, HPV43, HPV44, HPV55, HPV57, HPV74, HPV77, HPV91, HPV94, HPV117 and HPV125.

^b^Clinical specimens were tested using in-house test with primer set targeting the following HPV genotypes: HPV1, HPV2, HPV3, HPV10, HPV27, HPV28, HPV29, HPV41, HPV57, HPV63 and HPV77, as described previously [1], and Ma/Ha nested PCR system targeting at least 20 different cutaneotropic HPV types, as described previously [2, 3].

^c^Clinical specimens were tested with Linear Array HPV Genotyping Test (Roche Molecular Diagnostics, Pleasanton, CA), which detects 36 different HPV types: HPV6, HPV11, HPV16, HPV18, HPV26, HPV31, HPV33, HPV35, HPV39, HPV40, HPV42, HPV44, HPV45, HPV51, HPV52, HPV53, HPV54, HPV56, HPV58, HPV59, HPV61, HPV62, HPV64, HPV66, HPV67, HPV68, HPV69, HPV70, HPV71, HPV72, HPV73, HPV81, HPV82, HPV83, HPV84 and HPV89, and one HPV subtype: subHPV-82 or IS39.

^d^Clinical specimens were tested with RHA Kit Skin (beta) HPV (Diassay BV, Rijswijk, The Netherlands), which detects 25 different HPV types: HPV5, HPV8, HPV9, HPV12, HPV14, HPV15, HPV17, HPV19, HPV20, HPV21, HPV22, HPV23, HPV24, HPV25, HPV36, HPV37, HPV38, HPV47, HPV49, HPV75, HPV76, HPV80, HPV92, HPV93 and HPV96.

^e^Clinical specimens were tested with previously described CUT primer set, targeting at least 88 different cutaneotropic and mucosotropic HPV types [4].

NA; data not available.

**References**

[1] Shamanin V, Delius H, de Villiers EM. Development of a broad spectrum PCR assay for papillomaviruses and its application in screening lung cancer biopsies. J Gen Virol. 1994;75: 1149-1156.

[2] Boxman IL, Berkhout RJ, Mulder LH, Wolkers MC, Bouwes Bavinck JN, Vermeer BJ, et al. Detection of human papillomavirus DNA in plucked hairs from renal transplant recipients and healthy volunteers. J Invest Dermatol. 1997;108: 712-715.

[3] Boxman IL, Hogewoning A, Mulder LH, Bouwes Bavinck JN, Schegget J. Detection of human papillomavirus type 6 and 11 in pubic and perianal hair from patients with genital wart. J Clin Microbiol. 1999;37: 2270-2273.

[4] Chouhy D, Gorosito M, Sánchez A, Serra EC, Bergero A, Fernandez Bussy R, et al. New generic primer system targeting mucosal/genital and cutaneous human papillomaviruses leads to the characterization of HPV 115, a novel Beta-papillomavirus species 3. Virology. 2010;397: 205-216.
